# Revolutionizing pharmaceutical innovation: Unveiling the impact of endogenous knowledge spillover in China

**DOI:** 10.1371/journal.pone.0307171

**Published:** 2024-09-20

**Authors:** Zhuolin Li, Lin Guo, Ying Ping

**Affiliations:** College of Economics and Management, Shanghai Ocean University, Shanghai, China; Instituto Tecnologico Autonomo de Mexico, MEXICO

## Abstract

Modern medical technology innovation is a critical safeguard for human health, while a significant number of developing nations are confronted with the challenge of biopharmaceutical technological advancement. To investigate the possible routes of technological advancement, we investigated the impact of the endogenous knowledge spillover effect on firm innovation endeavors. Our research involves a theoretical two-stage R&D game model that is built around the characteristics of pharmaceutical organizations. Theoretical studies elucidated the impact as well as the probable mechanism of the spillover effect. To verify the theoretical study, we conducted econometric analysis using data from the pharmaceutical sector of Chinese enterprise listed on the A-share market. The study’s findings indicate that endogenous knowledge spillovers impede organizations’ innovation endeavors. This phenomenon may be attributed to the existence of the patent race paradigm and high concentration of enterprises’ R&D endeavors in specific areas. Additional examination of heterogeneity demonstrates that private firms, small and medium-sized enterprises (SMEs), and non-high-tech enterprises experience a larger adverse impact from the spillover effect. Hence, we suggest implementing "loser’s subsidies", reallocating R&D resources, and making modifications to competition policies as measures to enhance the innovation performance of biopharmaceutical markets. These policies will facilitate the technical advancement of medicines in developing nations.

## 1. Introduction

The advancement and utilization of life sciences and biotechnology have significantly transformed humanity’s comprehension of life essence, overhauled the prevention, diagnosis, and management of illnesses, and delivered unparalleled advancements in enhancing human health and well-being. In the context of China’s 14th Five-Year Plan, the biopharmaceutical industry has placed a high priority on the exploration and development of innovative medications, leading to an average annual growth rate of over 20% in research and development (R&D) investment across the sector. As a result, the number of new drugs under research and development has risen to the second position globally [1]. However, the current healthcare system in China is not adequately meeting the medical needs of the population, which presents a challenge for the country in terms of advancing biopharmaceutical innovation. The "2023 China Biopharmaceutical Industry Report" from the China Machinery Industry Information Research Institute revealed that only 9% of the domestic pharmaceutical market in China consists of innovative drugs, while other G20 countries have more than 20% of their markets dedicated to innovative drugs. Additionally, developed countries such as the United States, Japan, and Germany have over 50% of their pharmaceutical markets focused on innovative drugs [2]. This disparity in the production of high-end versus low-end drugs is impeding the realization of the "Healthy China" goal. Considering the situation in China, there is a need for comprehensive strategies to drive innovation in biomedical technology.

The knowledge spillover effect offers potential for improvements in technology across numerous developing nations during the early phases of drug innovation. These countries can avoid paying to invest in costly novel molecular entities but instead make quick technological development by imitating pharmacological therapy. Similar to other developing nations, China’s nascent pharmaceutical industry lacked the prerequisites for advanced pharmaceutical innovation due to its delayed inception, limited capital, inadequate accumulation of equipment and technology, unregulated market, and imperfect legal framework, leading it to adopt the strategy of imitating drug therapies to circumvent these challenges. Since the 1980s, domestic Chinese pharmaceutical firms have actively acquired foreign technologies and commenced the production of generic drugs [3]. As of 2021, the proportion of generic drugs in China is still as high as 63% [4]. (More detailed information regarding China’s biopharmaceutical industry development can be found in the online supplemental material). In the current phase of development, China’s pharmaceutical innovation advancement cannot rely solely on imitating drug therapies. Instead, it is necessary to reconsider the significance of knowledge spillover effects in achieving this upgrade.

The analysis of knowledge spillovers carries substantial economic and social implications and has attracted considerable attention from economists. Early studies concentrated on exploring the transfer effect, learning effect, and imitation effect associated with spillovers, and investigated the impact on productivity and creativity, leading to a significant amount of empirical evidence. The research had shown that knowledge spillovers have a substantial positive effect on the productivity and innovation persistence of German firms [5, 6], the productivity of the U.S. wartime shipbuilding industry [7], the creativity of India’s pharmaceutical manufacturing industry [8], and the knowledge acquisition capacity of start-ups [9]. Moreover, studies investigated the learning-by-hire effect (in the U.S. pharmaceutical industry) [10], the U.S. Patent Innovation Network [11], the Global Open-Source Software Project [12], and worldwide R&D mobility [13]. Their studies had verified the beneficial impact of knowledge spillover. Additionally, knowledge spillovers are recognized as a significant driver of economic growth in endogenous economic growth theory [14–16].

However, there is an increasing amount of evidence suggesting that spillover effects may not adhere to the idealized assumptions that were previously held, partly because competitive influences have been overlooked. Knowledge externalities refer to a range of factors, including the Marshall-Arrow-Romer (MAR) externality [17–19], the Jacobs externality [20], and the Porter externality [21], each of which has distinct impacts. When considering spillovers within competitive markets, they can lead to unforeseen conclusions. In academic settings, knowledge spillovers and competition effects may counteract each other, leading to a decline in academic output due to the law of diminishing marginal benefits [22]. Similar effects are observed in firm competition, where fierce competition discourages firms from innovating and results in an inverted U-shape relationship between spillovers and firm innovation [23, 24]. The positive effects of spillovers are limited by the fact that a large technological gap makes it difficult for laggards to absorb knowledge [25]. This article also investigates the spillover effects in competitive markets. Given that fierce competition can potentially distort the direction of R&D activities conducted by pharmaceutical companies [26], it is necessary to reassess the relevance of findings from previous research to the pharmaceutical market.

In addition, the complexity of spillover effects becomes more pronounced when the potential to respond to external knowledge is taken into account. Technology spillovers can occur naturally or be deliberately controlled, indicating that firms consider the influence of spillovers in their R&D decisions and strive to respond to knowledge externalities. The model of endogenous technological spillovers proposes that firms can respond to spillovers and enhance the benefits while minimizing the drawbacks by managing the turnover of R&D personnel [27]. The endogenous nature of spillovers is also evident in R&D collaboration between firms [28] and in firms’ patent transfer behavior in technology exchanges [29, 30]. However, it is regrettable that most of these studies are based on theoretical modeling explorations, necessitating the search for more empirical evidence.

Based on prior research, our investigation thoroughly explores the analysis of spillover effects, taking into account the impact of market competition and firms’ decisions to internalize spillovers. Specifically, we extend the foundational framework proposed by Bloom et al. [31], focusing on the characteristics of Chinese pharmaceutical firms. Our analysis centers on an R&D game involving N vertically integrated pharmaceutical firms, wherein firms initially decide on investments in the upstream R&D market before engaging in competition within the downstream product market. Throughout the game, pharmaceutical firms must consider the R&D investments and patenting activities of their rivals, utilizing patents to restrict knowledge spillovers. Ultimately, the equilibrium of a firm’s R&D investment hinges on the strategic substitution and strategic complementarity nature of the investment. To validate the practical applicability of these theoretical analyses, we compile a new dataset encompassing the financial information of Chinese A-share pharmaceutical listed companies and detailed patent application data, enabling a more precise assessment of endogenous knowledge spillovers.

This study presents interesting findings. Contrary to expectations, the research suggests that these spillovers may impede rather than enhance pharmaceutical innovation. This phenomenon occurs as a result of an abundance of pharmaceutical companies located in a single R&D area. It leads to a strategic substitution of investments in R&D among these companies, while operating within the limitations of the patent race paradigm. The empirical evidence supports these conclusions. The study recommends policy interventions such as "loser’s subsidies", reallocation of R&D resources, and adjustments to competition policies to encourage pharmaceutical firms to actively participate in R&D endeavors for the betterment of public health.

The potential contributions of this paper are as follows: Our study offers insights on the direction of technical advancement in emerging economies. In comparison to developed nations, the innovation system and policy environment in developing countries require enhancement, and spillovers do not operate within a perfect market mechanism. We examine the impact of spillovers using China, the world’s largest developing country, and propose potential routes to achieve high-quality innovation. Secondly, our study concentrates on endogenous knowledge spillovers and encompasses the competitive externalities of knowledge. This leads to distinctive findings and enhances the understanding of the relationship between spillovers and firm innovation. Notably, we find evidence that spillovers hinder innovative activity, aligning with existing theories of an inverted U-shaped relationship, particularly in the latter half of the curve. Thirdly, We created a novel dataset by acquiring comprehensive patent application information from Chinese biopharmaceutical companies through the PatSnap database. This data was then combined with existing financial data using precise field matching and fuzzy matching algorithms. This enables us to expand upon existing measures and investigate the impact of endogenous knowledge spillovers in greater depth.

The organization of this article is outlined as follows: Section 2 presents the analytical framework. Section 3 describes the data. Section 4 consists of empirical analysis, including validation of underlying assumptions, regression of benchmark models, robustness checks, mechanism analysis and heterogeneity. Section 5 outlines the policy implications. Section 6 summarizes and discusses the research findings.

## 2. Analytical framework

In this section, we initially examine a natural knowledge spillover scenario, where firms perceive knowledge spillovers as external factors and respond reactively to changes in external knowledge flows in their decision-making process. This natural knowledge spillover scenario serves as the fundamental framework for our subsequent analysis. Following this, we examine a knowledge protection scenario that is endogenously determined, in which the firm no longer perceives external knowledge as an exogenous variable. Instead, it exercises control over its interactions with external knowledge by engaging in patent filing.

### 2.1 Game settings

In both models, the game involves N firms, all of which are vertically integrated. The majority of Chinese pharmaceutical firms have adopted a vertically integrated organizational structure, a trend attributed by domestic Chinese scholars to the deficiencies in the Chinese market system. The high transaction costs associated with external transactions have led firms to internalize these costs, thus favoring vertical integration. Additionally, our model differs subtly from Bloom et al. [31] in that we incorporate the competitive impacts of knowledge spillovers without explicitly distinguishing between naturally occurring technological spillovers and those resulting from product competition. We argue that spillovers should include both forms, and our main concern is the final influence of spillovers on firms’ innovation endeavors.

In the game, all firms are arranged to engage in competition within both the upstream research and development (R&D) market and the downstream product market. Given that all firms are vertically integrated, the R&D decision-making process of each firm must take into account the goal of maximizing profits in the product market. Particularly, in the first stage, firms engage in R&D investment, with knowledge spillovers occurring between them. This implies that the R&D choices of a firm are influenced by the R&D decisions of its competitor firms. In the second stage, the firm competes in the downstream product market, which may involve quantity or price competition, without specifying the form of competition. It is crucial to acknowledge that increased investment in R&D can provide companies with a competitive advantage in product development, but it also entails additional expenses. Consequently, firms’ decisions in both R&D and product markets are interconnected, requiring them to consider how to maximize product market revenues when making R&D investments. In game modeling, we determine the game equilibrium using backward induction.

### 2.2 Natural knowledge spillover

First, let us analyze the competition in the product market (the second stage of the game). *π*_*i*_ = *π*(*r*_*i*_,*r*_−*i*_) is profit function for firm *i*, where *r*_*i*_ is the R&D investment of firm *i*. *r*_−*i*_ has a dual implication, as it refers to both the knowledge acquisition resulting from the natural transfer of technology and the strategic actions adopted by a corporation in reaction to the R&D activities of its rivals. Generally, *π*_*i*_ is increasing in *r*_*i*_ and concave but uncertain about *r*_−*i*_, i.e., $\frac{{\partial \pi }_{i}}{\partial r_{i}}>0,\;\frac{{{\partial \pi }_{i}}^{2}}{\partial^{2}r_{i}}<0,\;\frac{{\partial \pi }_{i}}{\partial r_{-i}}>\;or\;<0$, $\frac{{{\partial \pi }_{i}}^{2}}{r_{i}\partial r_{-i}}>\;or\;<0$. Note that firm *i*’s own R&D and other firms R&D are strategic substitutes if $\frac{{{\partial \pi }_{i}}^{2}}{{\partial r}_{i}\partial r_{-i}}<0$ and strategic complements if $\frac{{{\partial \pi }_{i}}^{2}}{{\partial r}_{i}\partial r_{-i}}>0$. In order to maximize profits in a competitive product market, the optimal response function for firm *i* is given by $x_{i}^{*}=argmax\;\pi (x_{i},x_{-i})$, $x_{i}^{*}$ could be the optimal output or price depends on whether firms compete on quantity or price. Solving for second stage Nash decisions yields $x_{i}^{*}=f(r_{i},r_{-i})$, we use $\Pi^{i}(r_{i},r_{-i})=\pi (x_{i}^{*},x_{-i}^{*})$ to represent the equilibrium of competition in the product market. For example, firms compete on quantity and *P* is the exogenous market price that firms are facing. Thus, the quantity *x* and the average cost *c* are the function of *r*_*i*_ and *r*_−*i*_, i.e., *x*(*r*_*i*_,*r*_−*i*_) or *c*(*r*_*i*_,*r*_−*i*_) [32]. If the firm profit in product market expresses as Π^*i*^ = *x*(*P*−*c*), thus Π^*i*^ is the function of *r*_*i*_ and *r*_−*i*_, i.e., Π^*i*^(*r*_*i*_,*r*_−*i*_).

After that, firm *i* selects optimal R&D expenditure to maximize net value *V*^*i*^ under unconstrained conditions:

$$\underset{r_{i}}{\max}\;V^{i}=\Pi^{i}(r_{i},r_{-i})-r_{i}$$ (1)

And the first order condition (FOC) is,

$$\frac{\partial V^{i}}{\partial r_{i}}=\Pi_{1}^{i}(r_{i},r_{-i})-1=0$$ (2)

where $\Pi_{1}^{i}=\frac{\partial \Pi^{i}}{\partial r_{i}}$. Similarly, $\Pi_{2}^{i}=\frac{\partial \Pi^{i}}{\partial r_{-i}}$, $\Pi_{22}^{i}=\frac{\partial {{(\Pi }^{i})}^{2}}{\partial^{2}{r-}_{i}}$. And $\Pi_{11}^{i}=\frac{\partial {{(\Pi }^{i})}^{2}}{\partial^{2}r_{i}}$ by the second order condition. Differentiating the formula:

$$\Pi_{11}^{i}dr_{i}+\Pi_{12}^{i}dr_{-i}=0$$ (3)

subsequently, we obtain:

$$\frac{dr_{i}^{*}}{dr_{-i}}=-\frac{\Pi_{12}^{i}}{\Pi_{11}^{i}}$$ (4)

The Eq (4) says that how *r*_−*i*_ affects $r_{i}^{*}$ depends on $\Pi_{12}^{i}$, because firms profit function is concave and thus $\Pi_{11}^{i}<0$. When *r*_*i*_ and *r*_−*i*_ are strategic substitutes, means $\Pi_{12}^{i}<0$ and make the $\frac{\partial r_{i}^{*}}{\partial r_{-i}}<0$. When firms R&D are strategic complements is similar, means $\Pi_{12}^{i}>0$ and make $\frac{\partial r_{i}^{*}}{\partial r_{-i}}>0$. It is straightforward to demonstrate that these conditions are applicable in the scenario involving the participation of N firms. In this fundamental game model, firms consider external knowledge as an exogenous factor and merely react passively to external knowledge. While the fundamental model may not entirely capture the complexity of firms’ R&D behavior, the equations offer a theoretical framework for comprehending firms’ R&D decisions.

### 2.3 Endogenous knowledge protection decisions

In a competitive market, companies consider the impact of knowledge spillovers on their R&D decision-making processes and employ various mechanisms to safeguard knowledge in order to capitalize on the economic benefits of technological innovations over an extended period. This illustrates the endogenous character of knowledge spillovers, indicating that organizations engage in knowledge management to protect their intellectual capital [27]. Enterprises employ various methods to safeguard their knowledge, such as intellectual property rights and technology secrecy, as suggested by Klein [33]. In the game, the patent application is depicted as the sole method for safeguarding knowledge. This study examines the decision-making process of firms in protecting their knowledge as an endogenous variable in their R&D decisions. Specifically, we expand upon the fundamental model and analyze a two-stage game.

In the first stage, firm *i* choose R&D investment *r*_*i*_ and the proportion of patent applications (or intensity of knowledge protection) *ρ*_*i*_∈[0,1]. Let *γ*∈[1,+∞) be the exogenous parameter represent the effectiveness of firms’ patent application behavior, its size depends on the quality of the intellectual property system in a given country or region and the characteristics of the industry’s knowledge production (e.g. R&D barriers). Thus, the rents that firm *i* can receive from technological innovations are *δ*_*i*_*k*^*i*^ = *k*^*i*^(*ρ*_*i*_*γ*+1−*ρ*_*i*_), *k*^*i*^ = *k*^*i*^(*r*_*i*_,*r*_−*i*_). *k*^*i*^ is increasing in both *r*_*i*_ and *r*_−*i*_ imply that $k_{1}^{i}>0$ and $k_{2}^{i}>0$. At the same time, patent application is not free of charge and incurs corresponding unit costs *g*, and the total cost of firm *i*’s patent application behavior is *gρ*_*i*_*k*^*i*^.

In the second stage, firms compete in product markets, similar to the previous basic model, the optimal response function for firm *i* is given by $x_{i}^{*}=argmax\pi (x_{i},x_{-i})$. In this function, $x_{i}^{*}$ could be the optimal output or price. The second stage Nash decisions yields $x_{i}^{*}=f(K_{i},K_{-i})$, where *K*_*i*_ = *δ*_*i*_*k*^*i*^ is firm *i*’s knowledge stock and *K*_−*i*_ = *δ*_−*i*_*k*^−*i*^ is other firms’ knowledge stock. And Π^*i*^(*K*_*i*_,*K*_−*i*_) is the equilibrium of competition in the product market. Note that Π^*i*^ is increasing in *K*_*i*_ and concave but decreasing in *K*_−*i*_, imply that $\Pi_{1}^{i}>0$, $\Pi_{11}^{i}$ <0 and $\Pi_{2}^{i}<0$. Based on the Nash equilibrium in the product market, we can solve for the optimal R&D investment $r_{i}^{*}$ and the optimal level of knowledge protection $\rho_{i}^{*}$. Specifically, firms make R&D decisions by maximizing Eq (5):

$$\underset{r_{i},\rho_{i}}{\max}\;V^{i}=\Pi^{i}(\delta_{i}k^{i}(r_{i},r_{-i}),\delta_{-i}k^{-i})-r_{i}-g\rho_{i}k^{i}(r_{i},r_{-i})$$ (5)

The first order conditions (FOC) are:

$$\frac{\partial V^{i}}{\partial r_{i}}=\delta_{i}\Pi_{1}^{i}k_{1}^{i}-g\rho_{i}k_{1}^{i}-1=0$$ (6)

$$\frac{\partial V^{i}}{\partial \rho_{i}}=(\gamma -1)\Pi_{1}^{i}k^{i}-gk^{i}=0$$ (7)

where $\Pi_{1}^{i}=\frac{\partial \Pi^{i}}{\partial {(\delta }_{i}k^{i})}$, $k_{1}^{i}=\frac{\partial k^{i}}{\partial r_{i}}$. Similarly, $k_{2}^{i}=\frac{\partial k^{i}}{\partial r_{-i}}$, $k_{22}^{i}=\frac{\partial {{(k}^{i})}^{2}}{\partial^{2}r_{-i}}$, $k_{11}^{i}=\frac{\partial {{(k}^{i})}^{2}}{\partial^{2}r_{i}}$, $k_{12}^{i}=\frac{\partial {{(k}^{i})}^{2}}{\partial r_{i}\partial r_{-i}}$. According to the FOC, we can obtain $\delta_{i}\Pi_{1}^{i}-g\rho_{i}=\frac{1}{k_{1}^{i}}$, $(\gamma -1)\Pi_{1}^{i}=g$. The total differential equations are obtained through the FOC:

(VririVriρiVρiriVρiρi)(dri*dρi*)=(−Vrir−idr−i−Vriρ−idρ−i−Vρir−idr−i−Vρiρ−idρ−i) (8)

In this paper, we focus on how R&D investment by other firms affects the R&D behavior of the focal firm *i*. By comparative static analysis based on multiple choice variables, we obtain:

$$\frac{dr_{i}^{*}}{dr_{-i}}=\frac{-V_{\rho_{i}\rho_{i}}V_{r_{i}r_{-i}}+V_{r_{i}\rho_{i}}V_{\rho_{i}r_{-i}}}{H}$$ (9)

$$\frac{d\rho_{i}^{*}}{dr_{-i}}=\frac{-{V_{r_{i}r_{i}}V}_{\rho_{i}r_{-i}}+{V_{\rho_{i}r_{i}}V}_{r_{i}r_{-i}}}{H}$$ (10)

$$\frac{dr_{i}^{*}}{d\rho_{-i}}=\frac{-V_{\rho_{i}\rho_{i}}V_{r_{i}\rho_{-i}}+V_{r_{i}\rho_{i}}V_{\rho_{i}\rho_{-i}}}{H}$$ (11)

$$\frac{d\rho_{i}^{*}}{d\rho_{-i}}=\frac{-{V_{r_{i}r_{i}}V}_{\rho_{i}\rho_{-i}}+V_{\rho_{i}r_{i}}V_{r_{i}\rho_{-i}}}{H}$$ (12)

When we observe the effect of *r*_−*i*_ on $r_{i}^{*}$, we assume that the change in *dρ*_−*i*_ is tiny. For the same reason, we assume *dr*_−*i*_ is tiny in a later study. In the above equation, $V_{r_{i}r_{-i}}=\frac{\partial {\left(V^{i}\right)}^{2}}{\partial r_{i}\partial r_{-i}}=\delta_{i}^{2}k_{1}^{i}k_{2}^{i}\Pi_{11}^{i}+\frac{k_{12}^{i}}{k_{1}^{i}}$, $V_{\rho_{i}r_{-i}}=\frac{\partial {\left(V^{i}\right)}^{2}}{\partial \rho_{i}\partial r_{-i}}=(\gamma -1)k^{i}k_{2}^{i}\Pi_{11}^{i}$, $V_{r_{i}\rho_{-i}}=\frac{\partial {\left(V^{i}\right)}^{2}}{\partial r_{i}\partial \rho_{-i}}=(\gamma -1){\delta_{i}k}_{1}^{i}k^{-i}\Pi_{12}^{i}$, $V_{\rho_{i}\rho_{-i}}=\frac{\partial {\left(V^{i}\right)}^{2}}{\partial \rho_{i}\partial \rho_{-i}}={\left(\gamma -1\right)}^{2}k^{i}k^{-i}\Pi_{12}^{i}$. $H=V_{r_{i}r_{i}}V_{\rho_{i}\rho_{i}}-V_{\rho_{i}r_{i}}V_{r_{i}\rho_{i}}>0$ is the hessian matrix. And $V_{r_{i}r_{i}}<0$, $V_{\rho_{i}\rho_{i}}<0$ because of concave net value function *V*^*i*^, $\Pi_{11}^{i}<0$ for the same reason. This means Π^*i*^ is a diminishing marginal return on *K*_*i*_. Note that the sign of $\Pi_{12}^{i}$ and $k_{12}^{i}$ is ambiguous. $V_{\rho_{i}r_{i}}{=V}_{r_{i}\rho_{i}}>0$ because more R&D leads to more knowledge protection. Based on the above analysis, it can be seen:

$$sign(\frac{dr_{i}^{*}}{dr_{-i}})=sign(k_{12}^{i})=\{\begin{array}{l} \;<0,\;k_{12}^{i}<0\\ ambiguous,\;k_{12}^{i}>0 \end{array}$$ (13)

Similarly, we have:

$$sign(\frac{d\rho_{i}^{*}}{dr_{-i}})=sign(k_{12}^{i})=\{\begin{array}{l} \;<0,\;k_{12}^{i}<0\\ ambiguous,\;k_{12}^{i}>0 \end{array}$$ (14)

And for the impact of *ρ*_−*i*_ on $r_{i}^{*}$ or $\rho_{i}^{*}$,

$$sign(\frac{dr_{i}^{*}}{d\rho_{-i}})=sign(\Pi_{12}^{i})=\{\begin{array}{l} <0,\;\Pi_{12}^{i}<0\\ >0,\;\Pi_{12}^{i}>0 \end{array}$$ (15)

$$sign(\frac{d\rho_{i}^{*}}{d\rho_{-i}})=sign(\Pi_{12}^{i})=\{\begin{array}{c} <0,\;\Pi_{12}^{i}<0\\ >0,\;\Pi_{12}^{i}>0 \end{array}$$ (16)

Similarly, we can obtain:

$$\frac{dk_{i}^{*}}{dk_{-i}}=\frac{-V_{\rho_{i}\rho_{i}}V_{k_{i}k_{-i}}+V_{k_{i}\rho_{i}}V_{\rho_{i}k_{-i}}}{H}$$ (17)

In the above equation, $V_{k_{i}k_{-i}}=\delta_{i}\delta_{-i}\Pi_{12}^{i},\;V_{\rho_{i}k_{-i}}=(\gamma -1){{\delta_{i}\delta_{-i}k}^{i}\Pi }_{12}^{i}$. Thus,

$$sign(\frac{dk_{i}^{*}}{dk_{-i}})=sign(\Pi_{12}^{i})=\{\begin{array}{l} <0,\;\Pi_{12}^{i}<0\\ >0,\;\Pi_{12}^{i}>0 \end{array}$$ (18)

These formulations suggest that the effect of spillovers on firms’ R&D behavior is contingent upon specific theoretical scenarios and assumptions. It is therefore logical to differentiate between the sign of $k_{12}^{i}$ and $\Pi_{12}^{i}$, specifically to examine two potential scenarios—strategic substitution ($k_{12}^{i}$ and $\Pi_{12}^{i}$ less than 0) or complementary ($k_{12}^{i}$ and $\Pi_{12}^{i}$ greater than 0) of R&D endeavors among enterprises.

Firstly, in the case where R&D endeavors among firms act as strategic substitutes, the R&D expenditures and patent application activities of competitors will diminish the optimal R&D investment and the inclination to seek patents for the firm (see Eq (13) to Eq (16)). This implies that the occurrence of spillovers in this situation will adversely affect the innovation activities of firms. The concept of strategic substitutes in investment can be elucidated by considering these analyses within the framework of a patent race. According to Loury’s patent race model [34], the initial firm that invests in R&D and obtain a patent will possess a distinct competitive edge. This edge is achieved by preventing rivals from acquiring comparable technology. Consequently, subsequent researchers may invest less in R&D, thereby reducing the probability of obtaining relevant patents, which leading to negative spillover effects [35].

Furthermore, the scenario becomes increasingly intricate when firms’ R&D endeavors exhibit strategic complementarity (also see Eq (13) and Eq (14)). This is attributed to our inability to ascertain the positive or negative nature of $V_{r_{i}r_{-i}}$. The strategic complementary of R&D investments between enterprises stems from the fact that organizations operating in the same technology field follow distinct R&D methods, enabling mutual learning among them [36]. Moreover, the technologies utilized vary to a certain degree, leading to research and development companies manufacturing items that are distinctive. As a result, these firms are able to establish separate market fields. This implies that there is a lack of severe competition between enterprises, or that the impact of business stealing effect is not considerable. In this scenario, the beneficial impacts of natural knowledge spillover prevail, and these effects generally have favorable benefits. The presence of strategic complementary in R&D among enterprises is a widespread phenomenon that presents a challenge for future empirical research.

Ultimately, the findings indicate that the strategic substitutions or complementarity of knowledge stock between firms will have an impact on the knowledge stock of the firm *i* (see Eq (18)). This is akin to the examination of R&D expenditures mentioned earlier.

Within the theoretical framework, we establish fundamental assumptions and differentiate between two potential scenarios. Next, we need to decide if R&D endeavors made by biopharmaceutical companies in China are strategic substitutes or strategic complements.

### 2.4 R&D activities in chinese biopharmaceuticals

Considering the fundamental facts and underlying reasons presented in the subsequent paragraphs, it is highly probable that R&D operations among Chinese pharmaceutical firms are strategic substitutes rather than strategic complements.

Firstly, the scenarios outlined in the patent model [34] closely resemble the R&D practices of biopharmaceutical companies. Owing to reduced R&D expenses associated with generic drugs, manufacturers of generic medications possess a competitive cost advantage. With an increasing number of companies entering the market to produce similar drugs, these entities are likely to provide greater discounts to consumers, resulting in a decline in overall market prices. This leads to significant profit losses for the original drug manufacturers, creating challenges in recovering their R&D costs [37]. Consequently, pharmaceutical companies are reluctant to disclose their technology, and there are significant financial motivations for them to utilize patents as a mean to constrain the R&D endeavors of their rivals. These practices discourage subsequent R&D activities, leading to a substitution of R&D investments [38]. Additionally, empirical evidence presented by Galasso [39] highlights the challenges of coordination between patent applicants and subsequent researchers in the pharmaceutical market.

In addition, let us consider the potential outcomes of firms opting for different research line. Given the divergence in R&D tracks among firms, the patenting activities of one firm do not impede the R&D efforts of others. Consequently, the R&D market could achieve greater economic efficiency. Regrettably, as highlighted by Kong et al. [40], Chinese pharmaceutical companies exhibit a relatively uniform product pipeline, with many companies concentrating on a single therapeutic area (oncology) and employing similar technologies. This has led to significant overlap in the research lines of pharmaceutical companies, resulting in a high level of substitution in their R&D investments. Consequently, Chinese biopharmaceutical companies are facing intense competition characterized by a lack of diversity in the market. Presently, the negative consequences of business stealing effect are amplified, while the beneficial impacts of natural spillover are reduced. This results in negative spillover consequences.

### 2.5 Hypotheses

To summarize the fundamental ideas of these theoretical analyses, we propose the following hypotheses:

Hypothesis 1 (H1): There exists strategic substitutes in R&D investments among Chinese biopharmaceutical companies.
Hypothesis 2 (H2): If R&D investments exhibit strategic substitutes, then endogenous knowledge spillover effects will hinder the improvement of innovation performance in biopharmaceutical companies.
Hypothesis 3 (H3): The patent race paradigm and excessive concentration in the research lines are sources of strategic substitutes in R&D strategies, exacerbating the negative impact of spillover effects on innovation activities of pharmaceutical companies.

It is evident that H1 serves as a premise, H2 posits hypotheses regarding the consequences of spillover effects, and H3 delves into the underlying reasons.

## 3. Data

### 3.1 Sample

We utilize data from Chinese biopharmaceutical A-share listed companies spanning the years 2005 to 2020 as the basis for our research. The financial information of these enterprises is sourced from CSMAR and CCER economic and financial databases, while their industry categorization is established in accordance with the 2021 version of industry classification standards by SHENYIN & WANGUO FUTURES Co., Ltd. Considering the time required for patents to be cited, the data is accurate as of the end of 2020. The patent data utilized in this study is sourced from the PatSnap Database, Google Patent Search System, and the Patent Search System offered by the China National Intellectual Property Administration (CNIPA).

During the patent information retrieval process, we employ the complete name of the company in both Chinese and English languages for conducting the search. The search encompassing patent offices across 164 countries (regions) globally, encompassing patents with legal statuses such as disclosure, substantive examination, authorization, before expiration of time limit, non-payment of the annual fee, restoration of the right, and partially invalid patents. It excludes patents with legal statuses of rejection, withdrawal, invalid application, abandonment, and other legal statuses.

In the procedure of aligning corporate financial data with patent data, we utilize the complete enterprise name as a distinctive identifier for the matching process. This involves employing a string-matching algorithm based on the Python 3.9 interpreter, enabling the integration of corporate financial data with patent data. Subsequently, the patent Pub. No. is employed as a unique identifier to link the original patent with its forward and backward citing patents. Ultimately, the aggregate count of patents identified amounts to approximately 683,555, encompassing both the primary patent data and its forward and backward citations.

Tables Z1-Z3 in S1 File presents the detailed patent information subsequent to the matching procedure.

### 3.2 Variables

#### 3.2.1 Patent activity

In accordance with Trajtenberg [41], employing citation-weighted patents as a surrogate measure for the patent activities (or innovation outputs) of a firm. *PatCited* is determined by adding the quantity of invention patents and utility model patents, as well as the quantity of their forward citations. And a greater number of citation-weighted patents indicates the increased economic and technological significance of the patents. Correspondingly, we can measure the patenting activity of other firms in the market and therefore define the variable *OPC*_*it*_ as the citation-weighted patents of other firms in the market.

#### 3.2.2 R&D stock (or intellectual capital)

Refer to the classical method of Hall [42] to calculate the firm’s research and development (R&D) stock:

$${RdStock}_{jt}={RD}_{jt}+(1-\delta ){RdStock}_{j,t-1}$$ (19)

where the rate of depreciation *δ* = 15% which aligns with the approach of Hall [42] and Pang et al. [43]. And initial firm R&D stock is defined as:

$${RdStock}_{j0}={RD}_{j0}/(g+\delta )$$ (20)

where *RD*_*j*0_ is the firm *j*’s R&D in the first year of the sample period, and *g* is the average growth rate of R&D investment in the sample period. From this, we can derive the R&D stock of the company for each year. We estimate the overall quantity of R&D stock within the sector but excluding firm *i*, i.e., $ln{ORS}_{it}=ln(\sum_{j=1}^{N-1}{RdStock}_{jt})(i\neq j)$, *lnRdStock*_*jt*_ indicates the R&D stock of a particular firm *j*. The identical rationale results in *lnORD*_*it*_ (= $ln(\sum_{j=1}^{N-1}{RD}_{jt})(i\neq j)$).

#### 3.2.3 Endogenous knowledge spillover

The portfolio of patents submitted by a company provides valuable insights into the company’s R&D technology emphasis and knowledge management strategies. We regard patenting as a mechanism through which firms can protect their knowledge. For instance, if a company seeks a patent for the treatment and prevention of breast cancer (IPC Classification No. A61K36/8905), it signifies that the company is engaged in R&D activities within the technical domain of A61K (thus delineating the technical scope of the company’s R&D endeavors), has achieved specific outcomes, and aims to safeguard these R&D outcomes from infringement by submitting a patent application (or aims to prevent competitors from exploiting these R&D outcomes for financial gain, which underscores the nature of endogenous knowledge spillovers).

In the computation of spillover effects, our approach is followed by the research conducted by the work of Jaffe et al. [44] and Lucking et al. [45]. The k-dimensional column vector *S*_*i*,*T*_= (*S*_*i*,1,*T*_,*S*_*i*,2,*T*_,*S*_*i*,3,*T*_,…,*S*_*i*,*k*,*T*_)′ represents the patent share matrix of firm *i* in all technology subclasses in the *T* period (2005–2020). In the formula, *S*_*i*,*k*,*T*_ indicates the share of patents of firm *i* in the technology subclass *k* in period *T*. We define *S*_*i*,*k*,*T*_ by calculating the ratio of the number of IPC codes (3-digit) to the total number of IPC codes (also 3-digit), where duplicate IPC codes in one patent were removed. Normalize the column vector *S*_*i*,*k*,*T*_ and then obtain *S*_*i*,*T*_, i.e., $s_{i,T}=\frac{S_{i,T}}{{{(S}_{i,T}^{\prime }S_{i,T})}^{\frac{1}{2}}}$. The technical proximity $\omega_{ji,T}^{Jaffe}$ of firm *i* and firms *j* is the dot product of their standardized patent share matrices. In the calculation process, in order to prevent the incorporation of self-funded R&D investment as an external source of knowledge transfer in the knowledge spillover matrix, we assume that a firm’s technological proximity to itself is 0 (i.e., $\omega_{ii,T}^{Jaffe}=0$). Finally, by multiplying the technological proximity between two firms by the intellectual capital stock of firm *j*, one spillover variable is obtained. There are various firms in the market, and we engage in a comparable mathematical procedure where we aggregate these values (see Eq (21)). In the context of regression analysis, the derived spillover variable is divided by a factor of 1000 in order to reduce the magnitude of the unit difference between the variables.

$${Spill}_{it}=\sum_{j=1}^{N-1}\omega_{ji,T}^{Jaffe}ln{RdStock}_{jt}$$ (21)

where $\omega_{ji,T}^{Jaffe}=s_{j,T}^{\prime }s_{i,T}(i\neq j)$.

In our analysis, we derive the technology proximity matrix by exclusively considering the patents filed by firms within the same industry as the focal firm. This approach incorporates competition effects into the assessment of spillovers, aligning with the theoretical framework we have developed.

#### 3.2.4 Technology gap

The technology gap (*TechGap*) refers to the disparity in total factor productivity between a specific firm and a leading-edge technology firm. The LP method, as proposed by Levinsohn and Petrin [46], is employed to compute the total factor productivity of all firms. Subsequently, the disparity in TFP between a specific firm and a technologically advanced firm is determined. Initially, as demonstrated in Eq (22), the production function is established as:

$$lnY_{it}=\alpha_{o}+\alpha_{1}lnK_{it}+\alpha_{2}lnL_{it}{+\alpha }_{3}lnM_{it}+\alpha_{4}ln{age}_{it}+\alpha_{5}{SOE}_{it}+\tau_{year}+v_{prov}+\varphi_{ind}+\epsilon_{it}$$ (22)

In the formula, *lnY*_*it*_、*lnK*_*it*_、*lnL*_*it*_、*lnM*_*it*_ are the natural logarithm of the actual operating income, net fixed assets, number of employees and intermediate input of the enterprise respectively. And *SOE*_*it*_ is a dummy variable indicates the nature of the property right (if it is a state-owned enterprise, the value is 1, otherwise the value is 0), *τ*_*year*_, *υ*_*prov*_, *φ*_*ind*_ are fixed effects of year, province and industry respectively. In the computation, the operating income is adjusted using the producer price index for pharmaceutical industrial products. The intermediate input is represented as the sum of operating costs and selling, administrative, and financial expenses, then reduced by the depreciation and amortization in the current period and the cash disbursed to employees. By estimating Eq (22), we can obtain the TFP of each firm. Further, the concept of the technology gap is delineated as stated by Aghion et al. [47]:

$${TechGap}_{it}=\frac{{TFP}_{mt}-{TFP}_{it}}{{TFP}_{mt}}(m\neq i)$$ (23)

$${TechGap}_{mt}=\frac{{TFP}_{mt}-{TFP}_{m-1,t}}{{TFP}_{mt}}$$ (24)

where *TFP*_*mt*_ is the largest TFP in the sample, and *TFP*_*it*_ is the TFP of firm *i* in the *t* period. If an enterprise is at the forefront of technology, the gap between the enterprise and the second largest *TFP*_*m*−1,*t*_ is used as the measurement standard for technology gap.

#### 3.2.5 Herfindahl-Hirschman Index

The Herfindahl-Hirschman Index (HHI) is frequently employed as a metric for assessing market structure or concentration. A higher HHI value indicates a more concentrated market. The calculation of HHI typically involves the use of operating revenue in Eq (25).

$${HHI}_{jt}=\sum_{i}^{N}{\left(\frac{Y_{ijt}}{X_{jt}}\right)}^{2}$$ (25)

where *HHI*_*jt*_ denotes the concentration of industry *j* in year *t*, *Y*_*ijt*_ denotes the operating income of firm *i* in industry *j* in year *t*, and *X*_*jt*_ denotes the sum of operating income of all firms in industry *j* in year *t*. $\frac{Y_{ijt}}{X_{jt}}$ denotes the market share of firm *i*. The HHI is calculated by summing the squares of the market shares of all enterprises in the market.

#### 3.2.6 Patent originality

The originality of a patent is determined by analyzing the backward citation information of the original patent [48]:

$${Origin}_{q}=1-\sum_{k=1}^{K}{\left(\frac{{BCited}_{qk}}{{BCited}_{q}}\right)}^{2}$$ (26)

where *BCited*_*q*_ is the total number of backward citations of patent *q*, and *BCited*_*qk*_ is the number of backward citations of the patent in the technology subclass *k*. The latter item in the formula reflects the concentration of backward citations of a patent, and generally speaking, the lower the concentration, the higher the originality of the patent. Patent originality (*Origin*) at the firm level is the average of all patents filed by firm *i* in period *t*.


$${Origin}_{it}=\sum_{q=1}^{Q}{Origin}_{itq}/{Pat}_{it}$$ (27)

The greater the patent originality index, the more receptive the organization is to assimilating knowledge from patents and other domains, and leveraging diverse knowledge combinations to drive innovation in the development of patented inventions. Calculating the mean value of patent originality for a firm is a straightforward and efficient method. In our way of calculation, the patent originality data we gained has a bimodal distribution, which means that one peak is close to zero and the other peak is greater than zero. The median or plurality inadequately captures the general situation in the presence of this data distribution. Averaging can account for all samples, even if certain contain extreme values; however, excessive bias can be substantially avoided through the implementation of tail-shrinking in data processing.

### 3.3 Descriptive statistics

In order to guarantee the accuracy and comprehensiveness of the data, the research samples are managed in the following manner: Samples with listing status of ST, *ST, suspended, or delisted in the present year are omitted. Furthermore, samples with incomplete observations for more than three consecutive years are also excluded. For instance, in the case of a specific enterprise variable, if the value of said variable is absent for three consecutive years, the data pertaining to the enterprise for these three consecutive years is omitted from the sample. We employ a comparable screening process for all variables. Utilizing the random forest algorithm, multiple imputation was conducted to select a dataset that maintains the original data distribution and exhibits a mean change of less than 5%. This approach aims to uphold the utmost integrity and accuracy of the data for analysis. All continuous variables undergo a 1% shrink-tail adjustment before and after to mitigate the impact of outliers on the empirical findings. Following this adjustment, a final unbalanced panel data set comprising 2,863 firm-year observations for 341 A-share listed companies is derived.

The variable names and symbols utilized in this paper are explicitly described in Table 1.The descriptive statistics of the variables discussed in this study are presented in Table 2. Detailed definitions of variables can be found in the Table Z4 in S1 File. The data utilized in this study, along with the regression code developed, are available in S1 Data.

**Table 1:** Variable names and symbols.

Variable Names	Variable Code
**Corporate investment in R&D**	*lnRD*
**Intellectual capital**	*lnRdStock*
**Competitors’ R&D investment**	*lnORD*
**Competitors’ intellectual capital**	*lnORS*
**Competitors’ citation-weighted patents**	*OPV*
**Adjusted operating income**	*lnY*
**Patents**	*Pat*
**Citation-weighted patents**	*PatCited*
**Endogenous knowledge spillover**	*Spill*
**Asset-liability ratio**	*Lev*
**The scale of enterprise**	*Scale*
**Nature of enterprise ownership**	*SOE*
**Return on assets**	*ROA*
**Total assets turnover**	*Turnover*
**Herfindahl-Hirschman index**	*HHI*
**Technology gap**	*TechGap*
**Patent originality index**	*Origin*

**Table 2:** Descriptive statistics.

Variables	Count	Mean	SD^①^	Min	Median	Max
***lnRD***	2863	17.40	1.360	13.24	17.42	20.51
***lnRdStock***	2863	19	1.310	10.14	19.02	23.37
***lnORD***	2863	23.50	0.820	21.69	23.63	24.56
***lnORS***	2863	25.08	0.420	24.40	25.01	25.82
***OPC***	2863	17937	4968	4022	19862	21888
***lnY***	2863	20.97	1.250	18.36	20.83	24.26
***Pat***	2863	33.33	63.04	0	14	844
***PatCited***	2863	84.07	138.6	0	38	1736
***Spill***	2863	2.400	1.090	0	2.440	4.490
***Lev***	2863	0.350	0.200	0.0400	0.320	0.870
***Scale***	2863	1.310	0.500	1	1	3
***SOE***	2863	0.260	0.440	0	0	1
***ROA***	2863	0.060	0.060	-0.180	0.060	0.240
***Turnover***	2863	0.610	0.360	0.120	0.530	2.070
***HHI***	2863	0.080	0.150	0.020	0.040	1
***TechGap***	2863	0.350	0.060	0.0300	0.360	0.750
***Origin***	2863	0.220	0.170	0	0.210	0.780

^**①**^ Standard deviation.

Table 2 provides basic information regarding the sample of enterprises. With respect to firm size, the sample comprises over 50% large-scale enterprises and a minority small and medium-sized enterprises, according to the firm size criteria established by the Chinese government. This is because our firm information primarily comes from publicly traded corporations, all of which have certain size. Regarding the nature of enterprise ownership, over 50% of the enterprises in the sample are privately owned, while a small number are state-owned. From a perspective of enterprise profitability, the majority of enterprises have a positive return on assets (*ROA*), which suggests that they are able to efficiently utilize their assets for profitable endeavors. Only a small minority of enterprises have a negative *ROA*. In terms of enterprise R&D investment, all sampled enterprises make annual investments in R&D, and the standard deviation of the pertinent variables reveals clear disparities between the magnitude of R&D investment and the stock of intellectual capital. When it comes to firms’ patent applications, there is a significant disparity in their capacity to obtain patents. The majority of enterprises may only submit applications for fewer than 15 patents annually, while a very small fraction can apply for more than 800 patents. Likewise, there exists a significant disparity in the quality and originality of patents. This can be observed in the significant variability of variables such as *PatCited* and *Origin*, as indicated by their substantial standard deviations. Concerning knowledge spillovers, the magnitude in the spillover variable from 0 to 4.49 indicates that there are substantial differences between organizations in terms of spillovers. Simultaneously, it exhibits a moderate standard deviation, further confirming the considerable disparity in spillovers among enterprises.

## 4. Empirical analysis

### 4.1 Assessing preconditions: Is there a potential for substitution in R&D among companies?

To initiate the empirical analysis, we begin by assessing the validity of H1. Specifically, we establish four equations as a foundation for this assessment. Eq (28), Eq (29), and Eq (30) investigate the potential substitution or complementary between research and development (R&D) investment, intellectual capital, and patent applications within firms, while Eq (31) examines the influence of competitors’ R&D investment on the change in operating income of a specific firm. Upon closer examination, these equations are interconnected and collectively depict the line of a firm from R&D to product sales. In the regression analysis, the coefficients of interest are denoted as $\beta_{0}^{r}$, $\beta_{1}^{s}$, $\beta_{1}^{p}$ and $\beta_{1}^{y}$.


$${lnRD}_{it}=\beta_{0}^{r}+\beta_{1}^{r}{lnORD}_{it}+\beta_{k}^{r}X_{it}+\phi_{ijt}+\varepsilon_{it}^{r}$$ (28)

$$ln{RdStock}_{it}=\beta_{0}^{s}+\beta_{1}^{s}ln{ORS}_{it}+\beta_{k}^{s}X_{it}+\phi_{ijt}+\varepsilon_{it}^{s}$$ (29)

$$g({PatCited}_{it})=\beta_{0}^{p}+{\beta_{1}^{p}OPC}_{it}+\beta_{k}^{p}X_{it}+\phi_{ijt}+\varepsilon_{it}^{p}$$ (30)

$${lnY}_{it}=\beta_{0}^{y}+\beta_{1}^{y}{lnORD}_{it}+\beta_{k}^{y}X_{it}+\phi_{ijt}+\varepsilon_{it}^{y}$$ (31)

When selecting control variables, we select variables that indicate characteristics that might potentially affect the R&D activities and patent application behavior of the organization. Specifically, we control the scale of the enterprise (*scale*) [49], the nature of the property (*SOE*), debt asset ratio (*Lev*), return on assets (*ROA*), circulating rate of total asset (*Turnover*), and the external competitive environment (*HHI*). At the same time, the fixed effect of firm (*η*_*i*_), year (*τ*_*year*_) and industry (*φ*_*Ind*_) is also controlled in the regression. To simplify the expression of the equations, we use *ϕ*_*ijt*_ to represent these three fixed effects. We argue that incorporating several fixed effects and adding control variables from a multidimensional perspective can assist us in mitigating the problem of endogeneity.

The results of the regression analysis are presented in Table 3. The estimation of coefficient $\beta_{1}^{r}$ indicates a significant strategic substitution of R&D investment among firms, with a statistically significant impact at the 1% level (see Table 3 Column (1)). The analysis reveals a strong investment substitution, with a 1% increase in total industry investment leading to a 50.7% reduction in the investment of a specific firm, suggesting a substantial effect. If the presence of investment from competing firms leads to a decrease in the investment made by a particular firm, it will also result in a reduction in the firm’s stock of intellectual capital and patent output. Table 3 Columns (2), (3) provide additional evidence supporting the robustness of this finding, showing that the stock of intellectual capital and patent applications of other firms significantly suppresses the relevant output of a given firm at the 5% and 1% significance levels, respectively. Additionally, Table 3 Column (4) demonstrates that R&D investment by other firms diminishes firm-specific operating income. These results are consistent with theoretical expectations and provide evidence for the existence of R&D investment substitutions.

**Table 3:** Results of assessing preconditions.

Variables	Estimated Coefficients			
	(1)	(2)	(3)	(4)
	Estimated enterprise R&D investment	Estimated enterprise intellectual capital	Estimated enterprise citation-weighted patents	Estimated enterprise revenues
***lnORD***	-50.745***			-4.135***
	(15.525)			(1.198)
***lnORS***		-34.489**		
		(14.886)		
***OPC***			-0.003***	
			(0.000)	
***Lev***	0.364	0.918***	0.287*	0.793***
	(0.276)	(0.230)	(0.160)	(0.153)
***Scale***	-0.388***	-0.131	-0.092	-0.290***
	(0.079)	(0.083)	(0.057)	(0.047)
***SOE***	-0.270*	-0.185	-0.169	0.117*
	(0.160)	(0.181)	(0.109)	(0.068)
***ROA***	0.753	0.589	0.091	1.731***
	(0.468)	(0.406)	(0.359)	(0.247)
***Turnover***	-0.283**	-0.184	-0.018	0.798***
	(0.143)	(0.160)	(0.102)	(0.084)
***HHI***	-1.001*	-0.436	-1.262***	-0.799***
	(0.555)	(0.343)	(0.471)	(0.273)
**Constant**	1,226.109***	870.434**	50.975***	118.318***
	(369.574)	(367.983)	(6.476)	(28.530)
**Firm, Year & Industry FE**	YES	YES	YES	YES
**Observations**	2,863	2,863	2,767	2,863
**R-squared**	0.523	0.555	0.844	0.767

Note: i. Columns (1), (2), (4) report panel fixed effects models (FEM) estimate as indicated.

ii. Column (3) report high-dimensional fixed-effect Poisson pseudo-maximum likelihood method (PPMLHDFE) estimates as indicated. Therefore, column report the Pseudo R-squared.

iii. All the standard errors are clustered at the firm level. Standard errors in parentheses.

iv. ***, **, and * indicate statistical significance at the 1%, 5% and 10% level, respectively.

Naturally, endogeneity is still not entirely circumvented. To assess the influence of these problems on the results, we repeat the regression by incorporating lagging one period explanatory variables and show the results in the Table Z5 in S1 File. This can significantly improve the robustness of the results.

### 4.2 Baseline estimates and results

Theoretical analysis indicates that if H1 is valid, then H2 will also naturally be valid. A patent equation is developed to assess the validity of H2,

$$E(P_{it}|x_{it}^{\prime })=exp(\delta_{k}x_{it}^{\prime })$$ (32)

further transformations yield:

$$g({PatCited}_{it})=\alpha_{0}+\alpha_{1}{Spill}_{it}+\alpha_{k}X_{it}+\phi_{ijt}+\vartheta_{it}$$ (33)

*PatCited*_*it*_ represents the number of citation-weighted patents obtained by enterprise *i* in period *t*, which is the explained variable. *Spill*_*it*_ is the surrogate variable of the knowledge spillover effect, which is the main explanatory variable in our model. The remaining variables carry the same significance as previously described. For estimation, we use the high-dimensional fixed-effect Poisson pseudo-maximum likelihood method (PPMLHDFE).

It is important to note that a commonly used estimation method involves taking the logarithm of the sum of the number of patents or citation-weighted patents plus one, and then conducting linear regression (“log one plus” regression). However, these models are prone to biased and inconsistent regression estimates due to the presence of heteroskedasticity, and may yield incorrect regression coefficient signs. Even when controlling for fixed effects, there is a likelihood of upward or downward bias, and the estimation coefficient of the zero value may lack practical economic implications [50]. In contrast, Poisson regression using pseudo-maximum likelihood estimation avoids these issues and makes fewer statistical assumptions about the distribution of the explained variables. Additionally, the accelerated HDFE–GIRLS algorithm enables the inclusion of multidimensional fixed effects in the model, thereby enhancing the efficiency of estimation [51]. In order to highlight the differences between the traditional method and the PPMLHDFE technique, we initially utilized the OLS method in the benchmark regressions, but later transitioned to using PPMLHDFE as the primary estimation method.

Table 4 shows the estimate results of these baseline models. Estimates of the patent equation suggest that the knowledge spillover will lead to a decrease in the quantity of patents weighted by citations, with a hazard ratio of 0.691 (*e*^−0.370^ = 0.691<1) or 0.677(*e*^−0.390^ = 0.677<1). This serves to validate the H2 (significant at 1% or 5%, Table 4, column (3) and (4)). Table 4 Columns (1) and (2) report OLS estimation, in line with standard methodology. The OLS regression results also validate the robustness of the conclusions. In conclusion, our analysis suggests that the spillover effects obstacle the innovation efforts of pharmaceutical firms, resulting in a decrease in the citation-weighed patents.

**Table 4:** Benchmark model regression results.

Variables	Estimated Coefficients			
	(1)	(2)	(3)	(4)
	Estimate using OLS without controls	Estimate using OLS with controls	Estimate using PPMLHDFE without controls	Estimate using PPMLHDFE with controls
***Spill***	-0.402***	-0.384***	-0.370**	-0.390***
	(0.114)	(0.115)	(0.157)	(0.147)
***Lev***		0.140		0.501**
		(0.216)		(0.235)
***scale***		-0.126		-0.136
		(0.079)		(0.094)
***SOE***		-0.266*		-0.208*
		(0.146)		(0.126)
***ROA***		0.541		0.409
		(0.487)		(0.504)
***Turnover***		-0.166		-0.041
		(0.142)		(0.165)
***HHI***		-2.331***		-1.601***
		(0.494)		(0.579)
**Constant**	6.306***	7.323***	5.979***	6.152***
	(0.806)	(0.900)	(0.379)	(0.467)
**Firm, Year & Industry FE**	YES	YES	YES	YES
**Observations**	2,863	2,863	2,767	2,767
**R-squared**	0.717	0.721	0.761	0.765

Note: i. Columns (1) and (2) report OLS estimates as indicated, and the explained variable is the natural logarithm of the citation-weighted patents plus one.

ii. Columns (3) and (4) report high-dimensional fixed-effect Poisson pseudo-maximum likelihood method (PPMLHDFE) estimates as indicated, correspond to Pseudo R-squared. The explained variable is the citation-weighted patents.

iii. When using PPMLHDFE estimates, some observations were deleted due to singletons [52]. Singletons are observations that have only one observation in the group, which if retained in the regression may exaggerate the significance of the regression coefficients and may lead to erroneous statistical inferences, and which Stata automatically removes in the regression.

iv. The standard errors are clustered at the firm level. Standard errors in parentheses, except for OLS regressions using robust standard errors.

v. ***, **, and * indicate statistical significance at the 1%, 5% and 10% level, respectively.

### 4.3 Robustness

#### 4.3.1 Endogeneity

The potential endogeneity in the patent equation can be attributed to three main factors: Firstly, there may be inaccuracies in measuring knowledge spillovers, with the inclusion of rent spillovers potentially leading to endogeneity issues in the model [53]. Secondly, the confusion of cause and effect may arise from external shocks or other confounding factors, such as positive external R&D shocks leading to an increase in R&D investment by all firms in the industry, which could be mistakenly attributed to the positive effects of knowledge spillovers [31]. Thirdly, the endogeneity of technological linkages between firms may raise concerns about the endogeneity of knowledge spillovers in the model [11]. Hence, in accordance with the study by Yang et al. [54], a shift-share instrumental variable (SSIV) was constructed and the baseline model was re-estimated using the control function method as outlined by Wooldridge [55].

The SSIV is computed by following the subsequent steps. Firstly, take year *t*_*o*_ as the base period and calculate the industry R&D growth rate in year *t* relative to year *t*_*o*_. After that, the R&D input of enterprises in each year is discounted according to the calculated industry R&D growth rate, and the R&D stock is further calculated. Finally, the original R&D matrix is replaced by the above-calculated-to R&D matrix during the calculation of the knowledge spillover proxy variables. The R&D industry growth rate is treated as an exogenous shock, which satisfies the exogeneity condition, and the initial value of R&D is the firm’s actual R&D input, which satisfies the relevance conditions.

The results of the regressions are presented in Table 5. The results of the estimation indicate that the issue of endogeneity is not of significant concern (note that the estimated coefficient on *v*2*h*_*fe* is not significant) in the patent equation and the above conclusions are robust.

**Table 5:** Regression results addressing model endogeneity.

Variables	Estimated Coefficients	
	(1)	(2)
	Stage 1	Stage 2
***Spill*_*IV***	0.983***	
	(0.009)	
***Spill***		-0.396***
		(0.149)
***v*2*h*_*fe***		-0.761
		(0.911)
**Constant**	0.017**	6.168***
	(0.007)	(0.472)
**Controls**	YES	YES
**Firm, Year & Industry FE**	YES	YES
**Observations**	2,863	2,767
**R-squared**	0.999	0.765

Note: i. Columns report control function method (CF) estimates as indicated.

ii. The first stage regression is ${Spill}_{it}={\alpha_{0}Spill}_{it}^{iv}+\alpha_{k}X_{it}+\eta_{i}+\tau_{year}+\varphi_{Ind}+\varepsilon_{it}$, we use panel fixed effects models (FEM) to estimate it. In the second stage, the obtained residuals with fixed effects are introduced into their respective regression equations, i.e., $g({PatCited}_{it})=\delta_{0}+\delta_{1}{Spill}_{it}+\delta_{k}X_{it}+v2h\_fe+\phi_{ijt}+\vartheta_{it}.v2h\_fe$ is the endogenous residual term obtained by the first stage regression. Column (2) report high-dimensional fixed-effect Poisson pseudo-maximum likelihood method (PPMLHDFE) estimates as indicated, correspond to Pseudo R-squared.

iii. Some observations were deleted due to singletons.

iv. All the standard errors are clustered at the firm level. Standard errors in parentheses.

v. ***, **, and * indicate statistical significance at the 1%, 5% and 10% level, respectively.

vi. The t-test results accept the null hypothesis that *Spill* is an exogenous variable.

#### 4.3.2 Additional robustness checks

Subsequently, we conducted various robustness tests to assess the reliability of the findings, and the regression results are detailed in the Tables Z6-Z8 in S1 File.

Considering government subsidies (*lnsub*), financing constraints (*KZ*) [56] and financial leverage (*FL*). Government subsidies plays a significant role in influencing firms’ decisions to innovate, as it provides crucial incentives for innovation [57, 58]. In order to assess the impact of these subsidies on innovation, we incorporate the government subsidy variable into the baseline equations. The regression findings indicate that government subsidies can enhance the number of citation-weighted patents held by firms at a statistically significant level of 5% or 10%, aligning with theoretical perspectives. It is important to highlight that the inclusion of subsidies in the regression does not alter the coefficients for *Spill*.

Brown et al. [59] assert that the R&D activities of young high-tech firms are influenced by cash flow and external equity. The presence of financing constraints is identified as a significant factor impacting R&D in high-tech firms. When incorporating the financing constraint variable into the regression analysis, the findings indicate that such constraints lead to an increase in firms’ R&D stock. This implies that heightened external financing constraints may prompt pharmaceutical companies to prioritize internal capital accumulation as an alternative to external financing in order to enhance R&D investment. It is noteworthy that the sign and magnitude of the primary explanatory variables of interest exhibit minimal change following the inclusion of the financing constraint variable. Furthermore, the coefficients of the primary explanatory variables remain largely unaffected even after the introduction of the financial leverage variable into the regression equation. Notably, financial leverage appears to have limited influence on corporate R&D behavior.

Redefine the explanatory variables. Given that the 3-digit IPC classification lacks precision and fails to adequately capture the diversity of patents within the category [60], we propose recalculating the technology proximity matrix using a 4-digit IPC in order to derive new variables (*Spill*4) for knowledge spillover in the patent equation. Furthermore, taking into account the impact of knowledge spillovers in relation to boundaries and distance [61–63], as well as the presence of cross-industry spillovers [64, 65], we propose a redefinition of two metrics for measuring knowledge spillovers. One approach involves mitigating the spillover effect based on distance (*techgeo*_*spill*), while the other aims to capture cross-industry spillovers through the analysis of forward and backward citations of the original patent (*inter*_*spill*). When altering the metrics of the primary explanatory factors, it is observed that knowledge spillovers continue to inhibit the increase in citation-weighted patents.

Redefine the explained variables. The citation-weighted patents may exhibit time-truncated impacts. We conducted additional regressions using the quantity of patents as the independent variable. The findings indicate that this adjustment does not alter the primary findings of the study.

Modify the configuration of the model form. First, we try to include more fixed effects in the benchmark models. As a result, the significance and size of the regression coefficients for the main explanatory variables remain virtually unchanged after we additionally include province as well as province & industry interaction fixed effects. Furthermore, we modify the clustering level by considering the interaction between year and industry. Subsequently, we also explore alternative regression methods including negative binomial regression, panel fixed effects regression, and panel Poisson regression. It is important to note that the fundamental findings of this study remain consistent despite variations in the model setup and regression techniques employed.

### 4.4 Mechanism

**Fig 1:**
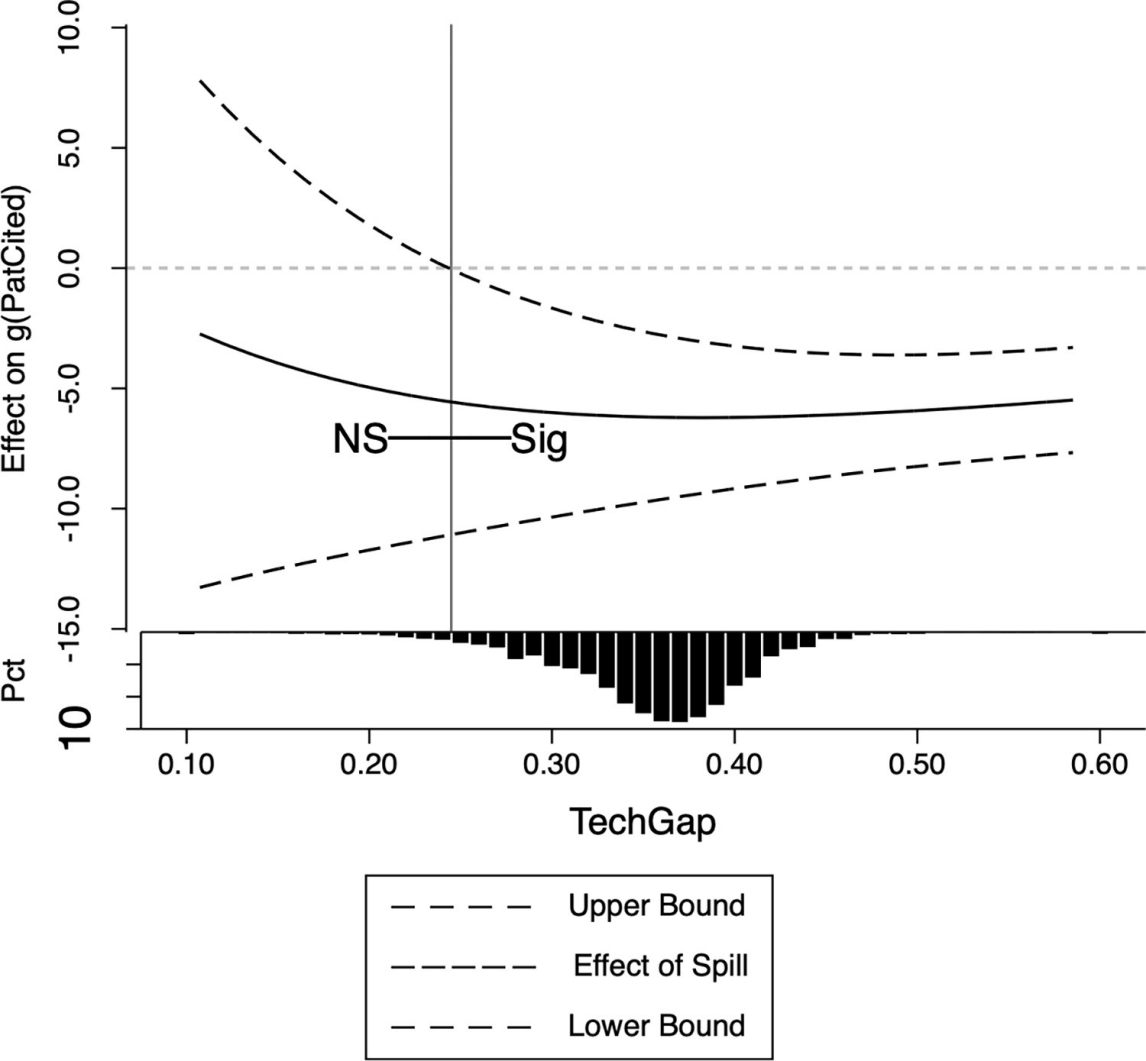
Moderating effects of the technology gap.

**Fig 2:**
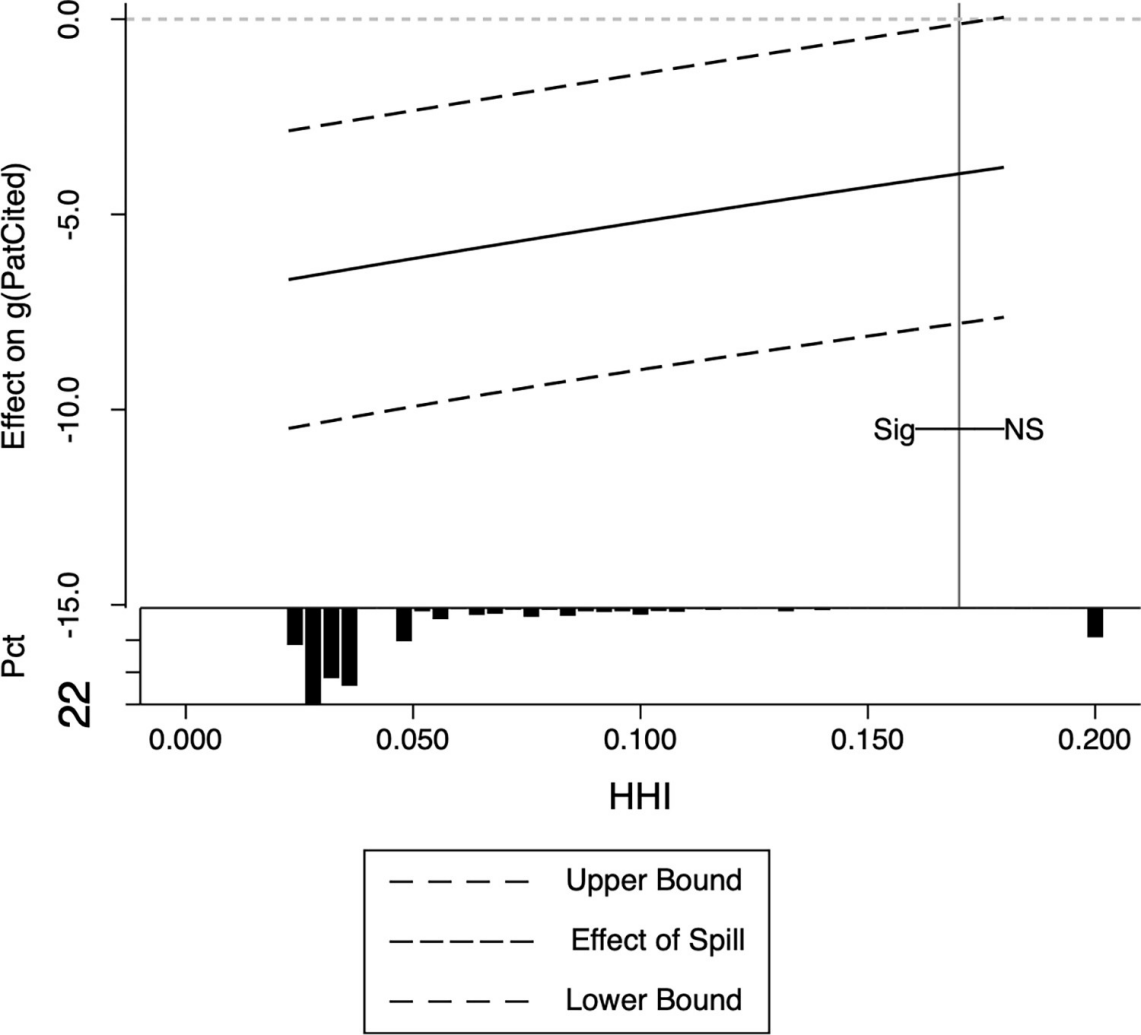
Moderating effects of market concentration.

**Fig 3:**
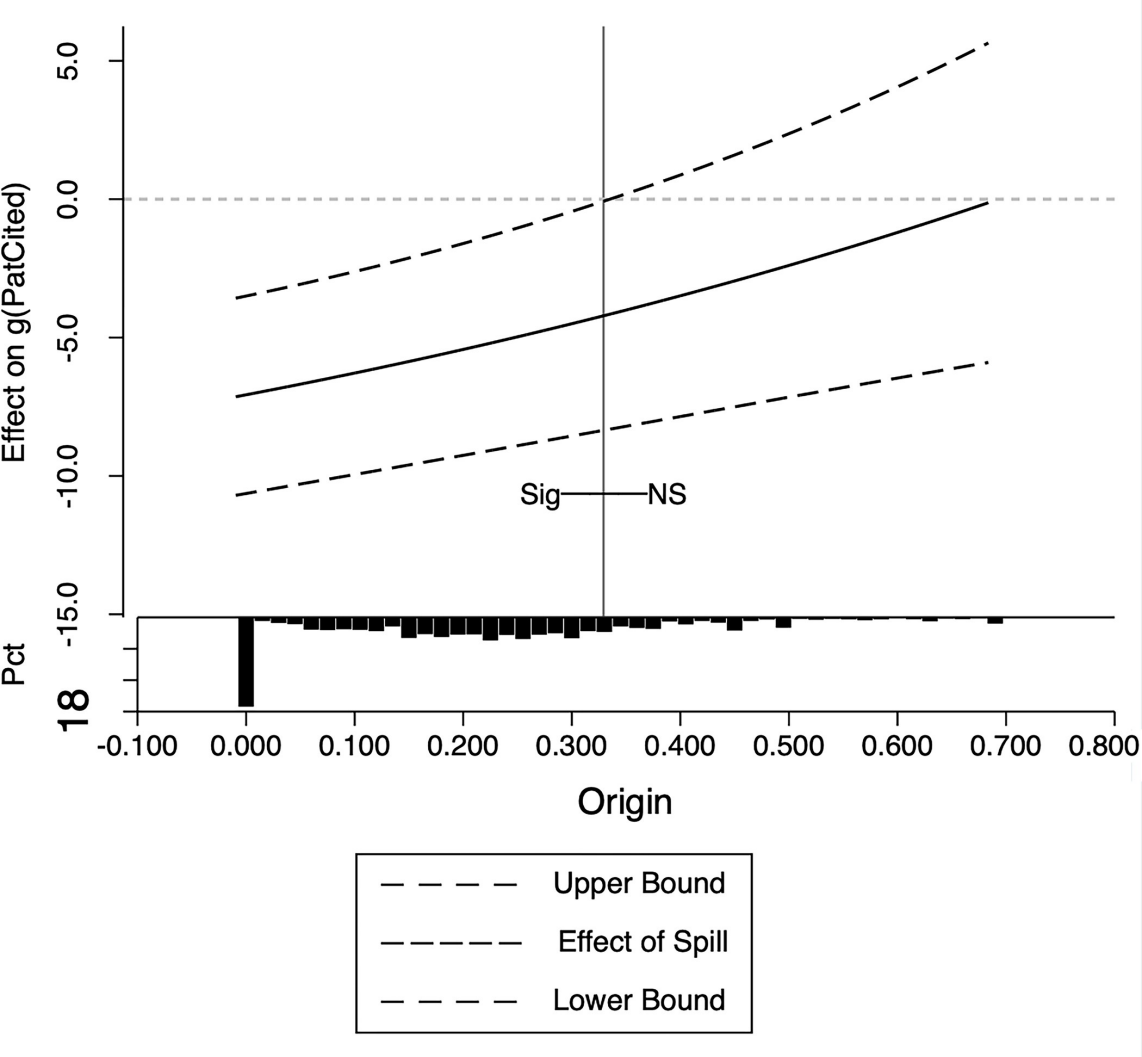
Moderating effects of enterprise patent originality. Note: In these pictures, the horizontal axis is the mechanism variable, and the vertical axis is the change of one standard deviation of the mechanism variable that brings about the change in *g*(*PatCited*_*it*_). The dashed lines in the figures show the upper and lower bounds of the confidence intervals, and the solid lines indicate the size of the spillover effects. The bottom histogram shows the distribution of moderating variables, with the vertical lines being the dividing lines between significant and non-significant, the labels NS being insignificant, and the labeling Sig being significant. If there is no vertical line, it means that it is significant in all intervals. All confidence levels in the figures default to 5%, and it was tested that using a higher confidence level (e.g. 1%) did not change the conclusions of this paper at all. The STATA program for picture production comes from Kaufman [66].

In our theoretical analysis, we posit that the strategic substitutions of R&D investments arise from the dynamics of patent races and excessive concentration in the R&D sector, leading to the dissemination of adverse spillover effects (as outlined in H3). As direct measurement of the R&D race pattern is challenging, we can infer the impact of the patent race (R&D competition) by assessing the technology gap between firms and the concentration of the product market. Additionally, due to limitations in available data, the specific research line chosen by each biopharmaceutical firm is not precisely known, however, we can speculate on whether firms pursue distinct research lines by examining the originality of the patents they file. To further investigate the reliability of the theoretical mechanism analysis, we elaborate on a moderated effects model.

$$g({PatCited}_{it})=\xi_{0}+\xi_{1}{Spill}_{it}\times {Mechan}_{it}+{\xi_{2}Mechan}_{it}+{\xi_{3}Spill}_{it}+\xi_{k}X_{it}+\phi_{ijt}+\varepsilon_{it}^{m}$$ (34)

where *Mechan*_*it*_ are the moderator variables, which can be *TechGap*,*HHI* or *Origin*. We also use the PPMLHDFE method for estimation. The estimates are presented in Table 6, while Figs 1–3 provide a detailed illustration of the influence of the moderator variables.

**Table 6:** Mechanism test results.

Variables	Estimated Coefficients		
	(1)	(2)	(3)
	Moderating role of the technology gap	Moderating role of the market concentration	Moderating role of the patent originality
***Spill***	0.062	-0.422***	-0.488***
	(0.212)	(0.147)	(0.158)
***TechGap***	0.897		
	(1.046)		
***HHI***	-1.549***	-3.414***	-1.647***
	(0.559)	(0.844)	(0.575)
***Origin***			-1.658***
			(0.411)
***TechGap*×*Spill***	-1.320***		
	(0.416)		
***HHI*×*Spill***		0.954***	
		(0.208)	
***Origin*×*Spill***			0.700***
			(0.157)
**Constant**	5.903***	6.216***	6.361***
	(0.644)	(0.466)	(0.488)
**Controls**	YES	YES	YES
**Firm, Year & Industry FE**	YES	YES	YES
**Observations**	2,767	2,767	2,767
**Pseudo R-squared**	0.768	0.767	0.768

Note: i. The explained variables in all regressions are citation-weighted patents.

ii. Columns report high-dimensional fixed-effect Poisson pseudo-maximum likelihood method (PPMLHDFE) estimates as indicated.

iii. Some observations were deleted due to singletons.

iv. All the standard errors are clustered at the firm level. Standard errors in parentheses.

v. ***, **, and * indicate statistical significance at the 1%, 5% and 10% level, respectively.

Table 6 Column (1) indicates a positive moderating impact of technology gaps on spillover effects. This suggests that companies with greater technology gaps will experience more adverse consequences from spillovers. Further elaboration is available in Fig 1. It has been observed that spillover effects have an insignificant impact on firms’ innovation endeavors when the technology gap is less than 0.274. This finding is based on a small subset of our data, and given the small technology gap, these firms are likely to be the industry leaders in terms of technology. Conversely, when the technology gap exceeds or equals 0.274, the adverse effects of spillovers become apparent, affecting the majority of firms. This suggests that the outcomes of R&D competition differ for successful and unsuccessful firms. The victors, namely the select few major technology companies with narrower technology gaps, are able to achieve initial success in R&D and secure patents. These firms exhibit minimal substitutes in their R&D investments and are relatively unaffected by knowledge spillovers. Conversely, the less successful entities, specifically the firms with wider technology gaps, are not as fortunate. They are unable to capitalize on R&D achievements and patent applications, and instead face reductions in R&D investment due to substitutions, as well as unrecoverable costs resulting from unsuccessful endeavors. These findings align with the theoretical analysis presented in the preceding section.

Table 6 column (2) shows that market concentration has a negative moderating effect on spillovers. This indicates that as market concentration decreases, indicating a more competitive market, the negative effects of spillovers become more pronounced. Conversely, when market concentration reaches or exceeds 0.878, spillovers cease to have a significant impact, and vice versa. (as depicted in Fig 2). Given the interrelation between the product market and the R&D market, as indicated in our theoretical analysis, heightened competition in the product market is likely to result in intensified competition in R&D. Consequently, it is plausible to posit that increased R&D competition facilitates the propagation of negative spillover effects. This further implies that R&D competition is a contributing factor to the observed negative impact of spillovers on innovative activity.

Table 6 column (3) shows that firms’ patent originality has a negative moderating effect on spillovers. This suggests that as a company’s patents become more unique, or as the company pursues a distinct research line, the negative spillover effect diminishes. Furthermore, the significance shifts when originality exceeds or equals 0.285 (as depicted in Fig 3). This implies that specific companies with higher originality can withstand the adverse effects of spillovers, which only represent a small portion of all firms in the sector. These analyses indicate that if a company is capable of pursuing a unique research line, it will experience minimal or no negative impact from spillovers. This underscores that the concentration of the research line is the underlying cause of the adverse impact of spillovers.

According to these analyses, we provide indirect confirmation that the patent race paradigm and the excessive concentration of R&D tracks are the underlying factors contributing to the spread of spillovers, thus supporting the validity of hypothesis H3.

### 4.5 Heterogeneity

In the benchmark model, it is assumed that spillover effects uniformly impact all firms. However, the theoretical analysis in Section 2 of this paper and the empirical tests in the preceding section indicate that the dynamics of patenting competitions, wherein some firms succeed while others do not, significantly contribute to the adverse consequences of spillovers. Specifically, firms that are unsuccessful in R&D competitions are more susceptible to the negative effects of spillovers. The identification of the firms that are likely to be unsuccessful in R&D competitions is crucial. We anticipate that the outcome of these competitions may be influenced by factors such as firm ownership, size, and technological characteristics. To investigate these variations, we conduct a re-estimation of the standard model using a divided sample.

Table 7 demonstrates the estimation results of the heterogeneity analysis. The estimation results in Table 7 Columns (1) and (2) differentiate the impact of firm ownership on the observed phenomena. The findings indicate that private firms (see column (1)) are more significantly influenced by negative spillover effects compared to state-owned firms (see column (2)). This disparity can be attributed to the financial backing provided to state-owned enterprises (SOEs) by the local or central government, their robust R&D capabilities, and their monopoly market power, which enables them to pursue multiple R&D directions to mitigate the risk of competitive failure. Furthermore, even in the event of failure, SOEs benefit from government support. In contrast, private firms lack these advantages and consequently face a higher risk of failure.

**Table 7:** Heterogeneity analysis results.

Variables	Estimated Coefficients					
	(1)	(2)	(3)	(4)	(5)	(6)
	Differentiating the nature of enterprise ownership		Differentiating enterprise scale		Differentiating the technical nature of enterprise	
***Spill***	-0.368**	-0.433	-0.334*	-0.771***	-0.508***	-0.192
	(0.184)	(0.285)	(0.202)	(0.296)	(0.182)	(0.186)
**Constant**	6.183***	5.915***	6.089***	6.085***	6.309***	5.366***
	(0.543)	(0.735)	(0.564)	(0.576)	(0.512)	(0.424)
**Controls**	YES	YES	YES	YES	YES	YES
**Firm, Year & Industry FE**	YES	YES	YES	YES	YES	YES
**Observations**	2,027	720	1,989	738	1,575	1,150
**Pseudo R-squared**	0.770	0.763	0.784	0.675	0.801	0.745

Note: i. The explained variables in all columns are citation-weighted patents.

ii. Columns (1) to (6) are subjected to regression analysis using data from privately owned businesses, state-owned enterprises (SOEs), large corporations, small and medium-sized enterprises (SMEs), non-high-technology companies and high-technology companies, respectively.

iii. Columns report high-dimensional fixed-effect Poisson pseudo-maximum likelihood method (PPMLHDFE) estimates as indicated.

iv. Some observations were deleted due to singletons.

v All the standard errors are clustered at the firm level. Standard errors in parentheses.

vi ***, **, and * indicate statistical significance at the 1%, 5% and 10% level, respectively.

The estimation results in Table 7 Columns (3) and (4) differentiate the impact of spillovers on enterprises of varying magnitudes. The findings indicate that small and medium-sized enterprises (SMEs) (see column (4)) experience a more pronounced and statistically significant negative impact from spillovers compared to large firms (see column (3)). The continuous nature of R&D activities holds crucial importance for pharmaceutical companies. Large firms have more stable funding sources for R&D, whereas SMEs have relatively limited funding options. Consequently, SMEs are at a financial disadvantage in competing with large firms and are more likely to suffer from the negative effects of spillovers, making them disadvantaged in the patent race.

The results of the estimation in Table 7 Columns (5) and (6) differentiate the technological characteristics of the firms. It is evident that high-tech firms (see column (6)) are less influenced by spillovers and have less significance compared to non-high-tech firms (see column (5)). High-tech firms engage in R&D activities, possess a well-established R&D infrastructure, exhibit greater R&D experience, and are likely to receive R&D subsidies from the government. These factors enhance the capacity of high-tech firms to assimilate knowledge [67, 68] and manage R&D risks more effectively. Conversely, non-high-tech firms lack these advantages, necessitating more exploration and R&D efforts to achieve similar R&D outcomes. Consequently, non-high-tech firms face a higher risk of R&D competitive failure and experience a more pronounced negative impact from spillovers. In summary, private enterprises, small and medium-sized enterprises (SMEs), and non-high-tech enterprises are disadvantaged in R&D competition and are adversely affected by spillover effects.

## 5. Policy implications

In this section, we analyze the policy implications arising from the findings presented in this paper. Our focus is on identifying the necessary mechanisms for enhancing the innovation performance of biomedical markets.

### 5.1 "Loser’s subsidies"

The patent race paradigm is identified as a fundamental contributor to distorting spillovers, which in turn discourages firms from engaging in innovation. It is important to note that the solution does not lie in the complete elimination of biomedical patents, nor does it entail excessively elevating patent protection. Rather, it is imperative to undertake essential reforms to the patent system. Initially, it is essential for research and development (R&D) subsidies and the patent system to work in conjunction with each other. The current patent framework results in private enterprises, small and medium-sized enterprises (SMEs), and non-high-tech enterprises being disadvantaged in the R&D competition, as they are affected by spillover effects and have weaker incentives for R&D. Consequently, it is imperative to implement a "loser’s subsidy" to address this issue. This subsidy would serve to reward firms engaged in R&D, irrespective of their success, with a specific focus on those with high R&D potential that have not yet achieved success. The objective is to nurture emerging innovative firms for future pharmaceutical innovation and to establish a conducive R&D environment. It is important to note that a loser’s subsidy does not entail a reduction in patenting standards or a bias towards technology-following firms. Acemoglu and Akcigit [69] contend that favoring technology-following firms in patent protection would lead to decreased innovation efficiency. Therefore, there is a need to guard the threshold for patenting to avoid the negative impact of " Bad money drives out good".

### 5.2 Reallocation of R&D resources

The excessive concentration of R&D activities in specific areas can also lead to distorted spillover effects. When a significant number of firms focus their R&D efforts in the same area, it can result in inefficiencies and may not be responsive to changes in market demand. To tackle these obstacles, it is imperative for governments to facilitate the market mechanism to ensure that the price mechanism accurately reflects shifts in market demand. In cases where there is an urgent need for certain medications or medical equipment, patients exhibit a greater willingness to pay, leading to higher market prices. Consequently, enterprises can adapt their R&D strategies and alter their R&D focus promptly in response to the pricing dynamics of their products in the pharmaceutical market, thereby preventing excessive concentration of enterprises in the R&D sector. On the contrary, the risk associated with R&D may serve as a significant deterrent for enterprises to transition to new research lines. Additionally, the investment in R&D fixed assets, considered as a sunk cost, may impede enterprises from venturing into new R&D avenues. State-owned firms, universities, research institutions, and other public sectors need to emphasize and strengthen their efforts in basic research. They should also actively participate in joint research and development initiatives with enterprises that are at the forefront of applied research. This approach aims to mitigate the risk associated with enterprise R&D and enhance innovation output [70, 71]. Furthermore, the government should consider implementing a system of accelerated depreciation of fixed assets and providing moderate subsidies for the procurement of R&D assets, which would contribute to enhancing industrial innovation performance [72].

### 5.3 Competition policy adjustments

In the theoretical analysis, it is observed that heightened market competition is associated with diminished innovation performance, particularly leading to the dissemination of spillover effects. In the context of pharmaceutical firms, the protracted R&D cycle and substantial capital outlay in biopharmaceuticals necessitate ensuring the sustained investment in R&D. Otherwise, prior efforts may be rendered futile. And corporate profits serve as a crucial funding source for corporate R&D endeavors. Consequently, it is imperative to establish a stable market competition environment, elevate the market access threshold moderately, and rigorously enforce the access system for pharmaceutical companies. On the other hand, it is essential to regulate the competitive conduct of businesses in order to prevent unfair competition and rent-seeking behavior, and to establish a standardized and orderly framework for market competition. This necessitates the implementation of anti-monopoly measures by the government to prohibit market position abuse, market action coordination, and monopolistic pricing. Additionally, it requires the enhancement of market competition regulations, and prohibition of unregulated drug production and sales practices. These systems and regulations hold significant importance in enhancing social welfare, as they can help rectify the imbalance between low-end surplus and high-end deficit in China’s pharmaceutical production, and ensure the safety of medicinal products, thereby safeguarding public health.

## 6. Conclusion and discussion

This study discusses the impact of endogenous knowledge spillovers on the innovation endeavors of biopharmaceutical companies. In competitive market settings, knowledge spillovers exhibit a "Janus face" effect, and the pharmaceutical innovation landscape in China is progressing towards the phase of original innovation, prompting a reevaluation of the implications of spillovers. To investigate these impacts, we construct a two-stage research and development (R&D) game model that permits interconnected competition in upstream R&D markets and downstream product markets, and necessitates that firms’ R&D investments must consider competitors’ R&D activities. Drawing on the outcomes of the game, we formulate research hypotheses and compile a novel dataset to examine the applicability of the theoretical analysis. In the section dedicated to econometric analysis, we assess whether the theoretical hypotheses align with empirical evidence and investigate the mechanisms and heterogeneity, subsequently proposing policy implications based on the findings. The findings of this research indicate:

- In competitive market environments, the impact of endogenous knowledge spillovers on the innovative endeavors of firms is contingent upon the strategic complementarity or substitutions of R&D investments among firms. When R&D investments among firms are strategically complementary, spillovers are likely to foster innovation. Conversely, in cases where they are strategic substitutes, spillovers are expected to deter firms from engaging in innovative activities.
- The strategic complementarity of R&D investments represents a broader scenario, and empirical studies often provide evidence that spillovers encourage firms to engage in innovation. Under specific circumstances, the strategic substitutions of R&D investments also apply. In the biopharmaceutical market, this substitution is driven by the competitive nature of patent races among firms and the over-concentration of corporate research lines, which can amplify the adverse effects of spillovers.
- Spillover effects have varying impacts on different types of enterprises. In particular, private firms, small and medium-sized enterprises (SMEs), and non-high-tech firms are adversely affected by spillovers. This is due to the higher likelihood of these firms failing in the R&D competition, resulting in increased substitutions of R&D investments under the patent race paradigm and consequently leading to greater negative spillover effects.
- In order to mitigate the negative effects of spillovers, several policy innovations are necessary. One such innovation is the implementation of a "loser’s subsidy" to foster a more permissive R&D environment and cultivate a new generation of innovators to enhance the industry’s innovation performance. Another necessary measure involves the redistribution of R&D resources to prevent the excessive concentration of R&D activities, mitigate intense homogenized competition, and rectify the imbalance of "low-end surplus and high-end insufficiency" in pharmaceutical supply. Additionally, adjustments to competition policy are essential to establish a favorable market competition environment and prevent the excessive erosion of profits for established enterprises by raising the market access threshold, thereby ensuring the quality of pharmaceutical supply.

Discussion of these results would be interesting. To the best of our knowledge, the notion that "knowledge spillovers motivate firms to innovate" is well supported by a number of researchers. For instance, Holl et al. [5] discovered that the local knowledge spillover has a beneficial effect on the firm’s constant innovation. Braunerhjelm et al. demonstrate that the spread of knowledge through the movement of workers promotes the innovation initiatives for companies [73]. Nevertheless, our research keeps a skeptical attitude towards these perspectives and asserts that specific market condition elements have the potential to disrupt the otherwise beneficial impacts of spillovers. Our research presents compelling evidence that the knowledge spillover in the biopharmaceutical market does not result in a significant increase in innovative activities. This might be linked to fierce competition within the market. The Chinese biopharmaceutical market is saturated with firms that concentrate on similar R&D fields and adopt parallel research lines. This results in fierce competition among firms, and it appears that the degree of competition does not contribute to the success of firms’ R&D efforts. We present empirical evidence that exhibits parallels with the findings of Audretsch and Belitski [24]. Their research discover a correlation between knowledge spillovers and corporate innovation that follows an inverted U-shaped pattern. They also identify market competition as a significant factor contributing to the inhibition of innovation caused by spillovers. Our empirical results are also consistent with the findings of Bryan et al. [26], who indicate that intense competition in the biomedical industry distorts the direction of R&D, leading to a reduction in social welfare.

Policies such as "loser subsidies" have been suggested in this paper as a way to enhance the innovation performance of the biopharmaceutical market in China. Nevertheless, it is imperative that we use prudence when implementing these policies. Firstly, their applicability is limited in extent. The biopharmaceutical market in China is experiencing an evolution, moving towards a phase of original innovation. The term "transition period" denotes a phase in which the industry’s focus is on enhancing the quality of innovations rather than only growing their quantity. Furthermore, the implementation of these policies necessitates an appropriate industrial framework, encompassing the construction of a modern pharmaceutical industry system, an existing institutional foundation, and a particular emphasis on pharmaceutical intellectual property rights. If the biopharmaceutical industry of a developing country is in its early stages, it may be more advantageous to adopt imitations policies, since they may speed the growth of innovative technologies. Market-oriented policies will be useless in a country that lacks the institutional foundation of a market. Beyond that, the implementation of policies to enhance the intensity of intellectual property rights (IPR) protection should be balanced to a country’s existing R&D capabilities. An excessive focus on IPR protection might hinder subsequent innovation, but a complete absence of safeguards for property rights is not conducive to significant advancements in innovation. In addition, in order to prevent false subsidies and stealing of subsidy funds by enterprises or R&D organizations, it is crucial to have a thorough understanding of the innovators when undertaking subsidy services.

Naturally, our study is subject to certain inherent constraints, which offer opportunities for further investigation. Primarily, our conclusions and policy suggestions are predicated on the premises of a competitive market and an imperfect patent system. Consequently, our research may have limited applicability in countries lacking a market-based system or where pharmaceutical patents are absent. It is advisable for developing nations in these circumstances to approach our findings with circumspection. Furthermore, despite the potential expansion of the existing data, it remains inadequate. Knowledge can be classified as either tacit or explicit, and references to patent literature typically only capture explicit knowledge exchanges, failing to adequately represent the transfer of tacit knowledge [74]. This issue highlights the need for future research endeavors. Subsequently, there is a necessity to develop improved methods for assessing tacit knowledge exchanges, which represents a significant area of interest for cutting-edge research. Finally, it is imperative for future studies to encompass a broader range of countries. The current paper lacks the ability to ascertain whether the findings are representative of the challenges faced by developing nations in general or are specific to the Chinese context. The ongoing investigation into pharmaceutical technology innovation underscores the necessity for additional empirical evidence to elucidate a trajectory for pharmaceutical technology advancement that aligns with the needs of the majority of developing countries.

## Supporting information

S1 File (DOCX)

S1 Data (ZIP)
