# Supplementary material for: Revolutionizing pharmaceutical innovation: Unveiling the impact of endogenous knowledge spillover in China
Source: PLoS One. 2024 Sep 20;19(9):e0307171. doi: 10.1371/journal.pone.0307171 (PMC11414994; doi:10.1371/journal.pone.0307171)
Supplement: S1 File — (DOCX) [file pone.0307171.s001.docx]

Supporting Information

# Industry Background

To put it simply, in the last 40 years, China's biopharmaceutical industry has gone from being a state of unorganized competition to exporting innovative medicines. The biopharmaceutical industry in China has followed a distinct trajectory due to a combination of factors, including but not limited to, internal governmental responses, changes in the external environment, and the evolution of principles and standards of economic development. This chapter presents a concise synopsis of the developmental history of China's biopharmaceutical industry, aiming to deepen readers' comprehension of China's unique circumstances.

Reform and opening up have presented substantial opportunities for the development of China's biopharmaceutical sector, which has been in progress since the 1980s. In 1980, the establishment of the first Sino-foreign joint venture medical company marked the beginning of foreign-funded pharmaceutical enterprises entering China. The Drug Administration Law of the People's Republic of China was adopted in 1984, allowing local governments to have some authority over drug approvals. During that period, drug approvals were primarily bureaucratic, with minimal technical evaluations, resulting in poor levels of regulatory efficiency and quality. The market was characterized by disorganized competition, with a prevalence of counterfeit and substandard pharmaceuticals. Since 1990, the State Council has been implementing measures to regulate the pharmaceutical sector, but initially, the impact was not notable. China's official entrance to the World Trade Organization (WTO) and subsequent full economic integration occurred in 2001, following the 1998 establishment of the National Medical Products Administration (NMPA). In the same year, the Drug Administration Law experienced its first systematic revision, which aimed to enhance the safeguarding of intellectual rights in order to satisfy the requirements of entering the WTO. Consequently, foreign enterprises saw a phase of rapid growth. The current version of the legislation also required the adoption of Good Manufacturing Practice (GMP) standards, which increased the minimum requirements for medication production and eventually phased out several outdated small and medium-sized businesses. In 2006, the State Council initiated an important effort to develop and produce novel drugs, with a specific focus on researching and developing innovative medicines. In 2010, the State Council identified the bio-industry as one of the seven critical emerging pillar industries. Currently, the national pharmaceutical market, primarily driven by generic medications, continues to face the issues of market confusion and inadequate oversight, resulting in inconsistent drug quality. In 2012, the NMPA began the process of Generic Drug Consistency Evaluation to assess the effectiveness of domestic generic drugs compared to their comparable original drugs. A protracted line of ambiguous definitions and inadequate oversight of generic medications has rendered the project continuing. During the period from 2015 to 2017, the government persistently implemented policies aimed at fostering pharmaceutical innovation. The "Made in China 2025" strategy, implemented in 2015, identified biopharmaceuticals as an essential industry. In the report of the 19th CPC National Congress in 2017, the government introduced a development strategy called "Healthy China." The strategy seeks to accelerate the growth of the health industry and enhance the medical and healthcare system. In 2020, the Patent Law was once more modified to provide further clarification on the process of resolving pharmaceutical patent conflicts through early settlement and compensation for patent term.

As of the present, China's biopharmaceutical industry has witnessed a substantial enhancement in its research and development (R&D) capacity. In 2023, China's medication research and development pipeline will account for 23.6% of the worldwide share, ranking second after the United States. A growing number of domestically developed novel pharmaceuticals are being introduced to the global market. Currently, China's biopharmaceutical industry is undergoing a shift from imitative innovation to original innovation, exhibiting numerous novel characteristics.

To begin within, in recent years, China has witnessed a more rapid expansion of its pharmaceutical R&D expenses. China's investment in pharmaceutical R&D has increased significantly from 14.3 billion yuan in 2017 to 31.9 billion yuan in 2021. This represents an average annual growth rate of over 20% over the previous five years, which is considerably greater than the United States' average growth rate of roughly 8.60% and that of other countries.

Furthermore, there is a pressing requirement for China's pharmaceutical industry to transition towards original innovation. As of August 2020, the proportion of preclinical, phase I clinical, phase II clinical, phase III clinical in China is 51.90%, 24.10%, 14.20%, and 7.60% respectively, according to publicly available data from the AVIC Research Institute. In comparison to the United States and the global average, China has a relatively lower proportion of preclinical studies (51.90% compared to 59.11% in the U.S. and 60% globally). However, China has a relatively higher proportion of Phase I clinical studies (24.10% compared to 18.05% in the U.S. and 16.40% globally) and Phase III clinical studies (7.60% compared to 6.00% in the U.S. and 6.10% globally). These findings indicate that China's biopharmaceutical R&D primarily emphasizes the development of established medications, while allocating fewer resources to the creation of novel inventions in the early stages.

Thirdly, China's drug R&D sector is highly concentrated and has a high degree of technical similarity. Statistics indicate that 47.57% of China's medication research and development (R&D) pipeline is dedicated to anti-cancer treatment. This sector has emerged as the leading focus of drug R&D in China, in terms of its proportion. Anti-infective and metabolic indications comprise the second and third largest indications for drug development in China, accounting for 9.07% and 8.09%, respectively. Regarding the concentration of mechanism of actions, China's TOP30 mechanism of action represented 45.36% of the whole, with a majority of pharmaceutical companies employing the same or similar mechanism of action. In comparison, the global average for the same period was 35.93%.

Plus, the level of competition in China's biopharmaceutical sector is intensifying. Based on data published by the National Bureau of Statistics of China, the quantity of above-scale biopharmaceutical enterprises (each with annual main business revenue of at least 20 million yuan) in the country increased to 9,231 as of 2022. In addition, since the outbreak of the Covid-19 epidemic, the average annual growth rate of these enterprises exceeded 5%. The introduction of these new elements in industrial development indicates that China's biopharmaceutical industry is currently experiencing a significant transformation, with a focus on enhancing the distinctive quality of pharmaceutical innovation as an essential direction.

# Detailed information on the patent applications of the companies in the sample

**Table Z1. Citations of patent applications submitted by enterprises**

|  | **Nonpatent**  **Backward Citations** | **Backward Citations** | **Forward Citations** | **Forward Citations**  **in 3 Years** | **Forward Citations**  **in 5 Years** |
| --- | --- | --- | --- | --- | --- |
| **count** | 105532 | 105532 | 105532 | 105532 | 105532 |
| **mean** | 0.833965 | 3.000739 | 1.376502 | 0.79248 | 1.051226 |
| **std** | 4.144792 | 9.594653 | 3.601276 | 2.192223 | 2.81795 |
| **min** | 0 | 0 | 0 | 0 | 0 |
| **25%** | 0 | 0 | 0 | 0 | 0 |
| **50%** | 0 | 1 | 0 | 0 | 0 |
| **75%** | 1 | 5 | 1 | 1 | 1 |
| **max** | 507 | 707 | 124 | 98 | 107 |

**Table Z2. Distribution of categories of patent applications submitted by enterprises**

| **Patent Type** | **Numbers** |
| --- | --- |
| **Invention patent application** | 45250 |
| **Utility model patent application** | 31352 |
| **Invention patent authorization** | 28930 |

**Table Z3. Distribution of IPC for patent applications by enterprises**

| **IPC (main)** | **Numbers** | **IPC (main)** | **Numbers** |
| --- | --- | --- | --- |
| **A61** | 30874 | B25 | 288 |
| **C07** | 26045 | B23 | 274 |
| **G01** | 10741 | A47 | 255 |
| **C12** | 6462 | F25 | 247 |
| **B65** | 6358 | F24 | 246 |
| **B01** | 4791 | C01 | 242 |
| **A01** | 1396 | H02 | 235 |
| **B08** | 1168 | B31 | 189 |
| **G06** | 1100 | E04 | 187 |
| **F26** | 1043 | H05 | 185 |
| **C08** | 1034 | B30 | 181 |
| **A23** | 944 | F28 | 165 |
| **B67** | 845 | A41 | 164 |
| **F16** | 720 | B41 | 162 |
| **B29** | 710 | B66 | 159 |
| **G16** | 637 | C03 | 157 |
| **H01** | 612 | B05 | 152 |
| **B02** | 471 | B04 | 147 |
| **H04** | 419 | B62 | 147 |
| **B26** | 416 | C09 | 137 |
| **B07** | 407 | C11 | 137 |
| **C02** | 386 | F21 | 122 |
| **F04** | 346 | G09 | 115 |
| **G05** | 345 | E05 | 112 |
| **G02** | 292 | E06 | 111 |

Note: The table displays the distribution of patents applied for by enterprises, limited by space. The calculations are based on the 3-digit IPC classification, and only the top 50 are shown.

**Variable definition**

**Table Z4. Variable definitions**

| **Variable categories** | **variable names** | **Variable Code** | **Variable Definition** |
| --- | --- | --- | --- |
| **Explained variables** | Citation-weighted patents | $PatCited$ | Number of invention patents + number of utility model patents + total number of forward citations to patents for inventions and utility models |
|  | Patents | $Pat$ | Number of invention patents + number of utility model patents |
|  | Intellectual capital | $lnRdStock$ | See Eq. (19), Eq. (20) in Section 3 |
|  | Adjusted operating income | $lnY$ | Operating income after deflating using price indices |
| **Main explanatory variables** | Competitors' research and development (R&D) investment | $lnORD$ | R&D of firms in the sample other than self |
|  | Competitors' intellectual capital | $lnORS$ | Intellectual capital of firms in the sample other than self |
|  | Endogenous knowledge spillover | $Spill$ | See Eq. (21) in Section 3 |
|  | Distance-weighted endogenous knowledge spillovers | $techgeo\_spill$ | See Eq. (c1) to Eq. (c3) |
|  | Cross-industry endogenous knowledge spillovers | $inter\_spill$ | See Eq. (c4) and Eq. (c5) |
| **Control variables** | The scale of enterprise | $scale$ | Large-scale enterprises = 1, medium-scale enterprises = 2, small-scale enterprises = 3, micro-enterprises = 4, refer to policy document for division |
|  | Nature of enterprise ownership | $SOE$ | State-owned enterprises = 1, private enterprises = 0 |
|  | Asset-liability ratio | $Lev$ | Total liabilities / Total assets |
|  | Return on assets | $ROA$ | Net profit / Total assets balance |
|  | Total assets turnover | $Turnover$ | Operating income / Total assets ending balance |
|  | Financing constraints KZ index | $KZ$ | Refer to Kaplan and Zingales [1] |
|  | financial leverage | $FL$ | (Net profit + income tax expense + finance costs) / (Net profit + income tax expense) |
|  | Government subsidy | $lnsub$ | ln (R&D subsidy + 1) |
| **Moderate variable** | Herfindahl-Hirschman Index | $HHI$ | See Eq. (25) in Section 3 |
|  | Technology gap | $TechGap$ | See Eq. (22), Eq. (23), Eq. (24) in Section 3 |
|  | Patent Originality Index | $Origin$ | See Eq. (26) and Eq. (27) in Section 3 |

# Results of robustness checks

**Table Z5. Regression results for solving endogeneity problems in preconditions regression**

| **Variables** | **Estimated Coefficients** | | | |
| --- | --- | --- | --- | --- |
|  | (1) | (2) | (3) | (4) |
|  | Estimated enterprise R&D investment | Estimated enterprise intellectual capital | Estimated enterprise citation-weighted patents | Estimated enterprise revenues |
| $\boldsymbol{L.lnORD}$ | -18.335*** |  |  | -3.635*** |
|  | (3.776) |  |  | (1.238) |
| $\boldsymbol{L.lnORS}$ |  | -29.678** |  |  |
|  |  | (12.558) |  |  |
| $\boldsymbol{L.OPC}$ |  |  | -0.001*** |  |
|  |  |  | (0.000) |  |
| $\boldsymbol{Lev}$ | 0.285 | 0.700*** | 0.167 | 0.738*** |
|  | (0.311) | (0.211) | (0.216) | (0.156) |
| $\boldsymbol{Scale}$ | -0.415*** | -0.081 | -0.078 | -0.319*** |
|  | (0.095) | (0.084) | (0.083) | (0.053) |
| $\boldsymbol{SOE}$ | -0.253 | -0.169 | -0.256** | 0.109* |
|  | (0.167) | (0.171) | (0.130) | (0.066) |
| $\boldsymbol{ROA}$ | 0.333 | 0.211 | 0.181 | 1.613*** |
|  | (0.495) | (0.374) | (0.433) | (0.242) |
| $\boldsymbol{Turnover}$ | -0.187 | -0.148 | -0.008 | 0.835*** |
|  | (0.151) | (0.153) | (0.143) | (0.088) |
| $\boldsymbol{HHI}$ | -1.155** | -0.393 | -1.800*** | -0.997*** |
|  | (0.567) | (0.315) | (0.548) | (0.237) |
| **Constant** | 454.937*** | 752.275** | 19.300*** | 106.584*** |
|  | (89.887) | (310.434) | (3.596) | (29.478) |
| **Firm, Year & Industry FE** | YES | YES | YES | YES |
| **Observations** | 2,521 | 2,521 | 2,459 | 2,521 |
| **(Pseudo) R-squared** | 0.383 | 0.560 | 0.780 | 0.762 |

Boundary and distance effects of knowledge spillovers. To account for the diminishing impact of knowledge spillover as distance increases, we operationalize the enterprise's specific location by utilizing the latitude and longitude coordinates of its headquarters office, and calculate the geographical distance of enterprise $i$ from all other firms $j$. Specifically, we examine a matrix representing the flow of knowledge, which diminishes in a linear fashion as the geographic distance increases, i.e.,

$$\begin{aligned} \omega_{ji,T}^{dis}=\frac{\max\left( D_{ji,T} \right)-d_{ji,T}}{\max\left( D_{ji,T} \right)}\#\left( c1 \right) \end{aligned}$$

$\max\left( D_{ji,T} \right)$ is the maximum value of the distance between all firms in the sample and $d_{ji,T}$ is the actual distance between firms 𝑖 and 𝑗. Notice that as $d_{ji,T}$ increases, $\omega_{ji,T}^{dis}$ will decrease. The distance-weighted technical proximity matrix is,

$$\begin{aligned} \omega_{ji,T}^{disaug}=\omega_{ji,T}^{Jaffe}*\omega_{ji,T}^{dis}\left( i\neq j \right)\#\left( c2 \right) \end{aligned}$$

Further, a distance-weighted knowledge spillover variable can be calculated,

$$\begin{aligned} {techgeo\_spill}_{it}=\sum_{j=1}^{N-1} \omega_{ji,T}^{disaug}ln{RdStock}_{jt}\#\left( c3 \right) \end{aligned}$$

Cross-industry spillovers. We utilize the citations from both forward and backward citations of the original patents to directly calculate the k-dimensional column vectors representing cross-industry spillovers $\omega_{ji,T}^{cites}'$. The distribution of the 3-IPC codes from the original patent citation is reflected in a weight matrix, which is utilized to weigh the Jaffe technology proximity matrix and subsequently standardized to derive $\omega_{ji,T}^{citesaug}$, i.e.,

$$\begin{aligned} \omega_{ji,T}^{citesaug}=\omega_{ji,T}^{Jaffe}*\omega_{ji,T}^{cites}\#\left( c4 \right) \end{aligned}$$

Further, it is possible to calculate the knowledge spillover matrix by taking into account both forward and backward citations,

$$\begin{aligned} {inter\_spill}_{it}=\sum_{j=1}^{N-1} \omega_{ji,T}^{citesaug}ln{RdStock}_{jt}\#\left( c5 \right) \end{aligned}$$

**Table Z6. Results of robustness tests with additional control variables**

| **Variables** | **Estimated Coefficients** | | | |
| --- | --- | --- | --- | --- |
|  | (1) | (2) | (3) | (4) |
|  | Consideration of government subsidies | Consideration of financing constraints | Consideration of financial leverage | Consideration of all three variables |
| $\boldsymbol{Spill}$ | -0.376** | -0.390*** | -0.387*** | -0.373** |
|  | (0.146) | (0.148) | (0.148) | (0.148) |
| $\boldsymbol{lnsub}$ | 0.021** |  |  | 0.021** |
|  | (0.008) |  |  | (0.008) |
| $\boldsymbol{KZ}$ |  | 0.000 |  | -0.000 |
|  |  | (0.011) |  | (0.011) |
| $\boldsymbol{FL}$ |  |  | -0.006 | -0.006 |
|  |  |  | (0.015) | (0.015) |
| **Constant** | 5.783*** | 6.153*** | 6.159*** | 5.789*** |
|  | (0.504) | (0.463) | (0.470) | (0.504) |
| **Controls** | YES | YES | YES | YES |
| **Firm, Year & Industry FE** | YES | YES | YES | YES |
| **Observations** | 2,767 | 2,767 | 2,767 | 2,767 |
| **Pseudo R-squared** | 0.766 | 0.765 | 0.765 | 0.766 |

Note: i. Proxy variables for government subsidies ($lnsub$), financing constraints ($KZ$), and financial leverage ($FL$) are added sequentially to the baseline regression, and ultimately all three variables are simultaneously incorporated.

ii. Columns report high-dimensional fixed-effect Poisson pseudo-maximum likelihood method (PPMLHDFE) estimates as indicated.

iii. Some observations were deleted due to singletons.

iv. All the standard errors are clustered at the firm level. Standard errors in parentheses.

v. ***, **, and * indicate statistical significance at the 1%, 5% and 10% level, respectively.

**Table Z7. Robustness check results with replacement of explanatory and explained variables**

| **Variables** | **Estimated Coefficients** | | | |
| --- | --- | --- | --- | --- |
|  | (1) | (2) | (3) | (4) |
| $\boldsymbol{Spill}\boldsymbol{4}$ | -0.414*** |  |  |  |
|  | (0.121) |  |  |  |
| $\boldsymbol{inter}\boldsymbol{\_}\boldsymbol{spill}$ |  | -0.578*** |  |  |
|  |  | (0.222) |  |  |
| $\boldsymbol{techgeo}\boldsymbol{\_}\boldsymbol{spill}$ |  |  | -0.376** |  |
|  |  |  | (0.157) |  |
| $\boldsymbol{Spill}$ |  |  |  | -0.403* |
|  |  |  |  | (0.213) |
| **Constant** | 5.933*** | 6.140*** | 6.046*** | 5.573*** |
|  | (0.354) | (0.489) | (0.457) | (0.673) |
| **Controls** | YES | YES | YES | YES |
| **Firm, Year & Industry FE** | YES | YES | YES | YES |
| **Observations** | 2,767 | 2,767 | 2,767 | 2,767 |
| **Pseudo R-squared** | 0.766 | 0.767 | 0.765 | 0.783 |

Note: i. Explained variables in columns (1) to (3) are citation-weighted patents, whereas in column (4) is patents.

ii. Columns report high-dimensional fixed-effect Poisson pseudo-maximum likelihood method (PPMLHDFE) estimates as indicated.

iii. Some observations were deleted due to singletons.

iv. All the standard errors are clustered at the firm level. Standard errors in parentheses.

v. ***, **, and * indicate statistical significance at the 1%, 5% and 10% level, respectively.

**Table Z8. Robustness check results for applying multiple models**

| **Variables** | **Estimated Coefficients** | | | | |
| --- | --- | --- | --- | --- | --- |
|  | (1) | (2) | (3) | (4) | (5) |
| $\boldsymbol{Spill}$ | -0.526** | -0.390*** | -0.541*** | -0.384*** | -0.352** |
|  | (0.216) | (0.119) | (0.140) | (0.125) | (0.145) |
| **Constant** | 6.511*** | 6.152*** | 9.267*** | 5.466*** | - |
|  | (0.595) | (0.344) | (1.394) | (0.426) |  |
| **Controls** | YES | YES | YES | YES | YES |
| **Firm, Year & Industry FE** | YES | YES | YES | YES | YES |
| **Observations** | 2,758 | 2,767 | 2,863 | 2,863 | 2,767 |
| **R-squared** | 0.768 | 0.765 | 0.112 | 0.078 | - |

Note: i. Column (1) add additional provinces, province-industry fixed effects.

ii. Column (2), The clustering level is year-industry level.

iii. Column (3), negative binomial regression (NB) estimates obtain an alpha value of 0.720, corresponding to a confidence interval [0.619, 0.837], which indicates the presence of overdispersion.

iv. Column (4) report panel fixed effects models (FEM) estimate as indicated, and the explained variable is the natural logarithm of the citation-weighted patents plus one.

v. Column (5) report Poisson estimate as indicated, and simultaneous use of ordinary robust standard errors.

vi. Some observations were deleted due to singletons.

vii. All the standard errors are clustered at the firm level (except for column 5). Standard errors in parentheses.

viii. ***, **, and * indicate statistical significance at the 1%, 5% and 10% level, respectively.

References

1. Kaplan SN, Zingales L. Do Investment-Cash Flow Sensitivities Provide Useful Measures of Financing Constraints?*. The Quarterly Journal of Economics. 1997;112(1):169-215.
